# Genome-Wide Survey and Expression Analysis of Amino Acid Transporter Gene Family in Rice (*Oryza sativa* L.)

**DOI:** 10.1371/journal.pone.0049210

**Published:** 2012-11-15

**Authors:** Heming Zhao, Haoli Ma, Li Yu, Xin Wang, Jie Zhao

**Affiliations:** State Key Laboratory of Hybrid Rice, College of Life Sciences, Wuhan University, Wuhan, China; Tel Aviv University, Israel

## Abstract

**Background:**

Amino acid transporters (AATs) that transport amino acids across cellular membranes are essential for plant growth and development. To date, a genome-wide overview of the *AAT* gene family in rice is not yet available.

**Methodology/Principal Findings:**

In this study, a total of 85 *AAT* genes were identified in rice genome and were classified into eleven distinct subfamilies based upon their sequence composition and phylogenetic relationship. A large number of *OsAAT* genes were expanded via gene duplication, 23 and 24 *OsAAT* genes were tandemly and segmentally duplicated, respectively. Comprehensive analyses were performed to investigate the expression profiles of *OsAAT* genes in various stages of vegetative and reproductive development by using data from EST, Microarrays, MPSS and Real-time PCR. Many *OsAAT* genes exhibited abundant and tissue-specific expression patterns. Moreover, 21 *OsAAT* genes were found to be differentially expressed under the treatments of abiotic stresses. Comparative analysis indicates that 26 *AAT* genes with close evolutionary relationships between rice and *Arabidopsis* exhibited similar expression patterns.

**Conclusions/Significance:**

This study will facilitate further studies on *OsAAT* family and provide useful clues for functional validation of *OsAATs*.

## Introduction

Amino acid transporters (AATs) are the integral membrane proteins which mediate the transport of amino acids across cellular membranes in higher plants, and play an indispensable role in various processes of plant growth and development, including long distance amino acid transport, response to pathogen and abiotic stresses [1]–[3]. By heterologous expression systems and database screening with known transporters, more than 60 distinct AAT genes have been identified in *Arabidopsis* [4], as well as several genes in other plant species.

Plant AATs family includes two main families that belong to the amino acid-polyamine- choline (APC) transporter superfamily: the amino acid/auxin permease (AAAP) family and the APC family [5], [6]. There are at least six subfamilies in the AAAP family, including amino acid permeases (AAPs), lysine and histidine transporters (LHTs), proline transporters (ProTs), γ-aminobutyric acid transporters (GATs), auxin transporters (AUXs), and aromatic and neutral amino acid transporters (ANTs). The APC family in plants is grouped into three subfamilies: cationic amino acid transporters (CATs), amino acid/choline transporters (ACTs) and polyamine H^+^-symporters (PHSs) [7]–[9].

Previously, molecular functions of many AATs were fully characterized in *Arabidopsis*. The eight members (*AtAAP1*-*AtAAP8*) were found in the *AtAAP* subfamily, and six AtAAPs were demonstrated to transport neutral and charged amino acids with varying specificities and affinities [10]–[12]. *AtAAP1* was highly expressed in the cotyledons and the endosperm and regulated the import of amino acid into root cells or developing embryo [13]–[15]. *AtAAP5* might transport amino acids into the developing embryo, and be the most important component in the root amino acid uptake system [16]. The analysis of *aap6* mutant demonstrated the physiological role of AtAAP6 in regulating sieve element composition [9]. *AtAAP8* might be required for amino acid uptake into the endosperm and developing embryos at early stages [17]. In addition, the functions of AAP gene members from other plant species had also been studied, such as *StAAP1* [18], *VfAAP1* and *VfAAP3* [19], *PvAAP1* [20]. Recently, it was proposed that *PtAAP11* played a major role in xylogenesis by providing proline in poplar [12].

AtLHT1, a specific transporter for lysine and histidine, was a key mediator of root uptake of amino acids and supply of leaf mesophyll with xylem-derived amino acids in *Arabidopsis* [21], [22]. AtLHT1 and AtAAP5 together played critical and separate roles in root uptake of cationic amino acids at concentrations typically possessed in soils [23], [24]. AtProTs were responsible for transporting proline, glycine betaine and GABA, and their expression patterns were complementary [25]. Although intracellular localization and substrate specificity were very similar, *AtProTs* fulfilled different roles in planta [26], [27]. It was reported that the rice ProT protein (OsProT) has 68.8% and 59.6% similarity to AtProT1 and LeProT1, respectively, and specifically transported L-Pro in a transport assay by using Xenopus laevis oocytes [28], [29]. In addition, heterologous expression analysis of *HvProT* indicated that it might play a crucial role in the transport of proline to root tip region and involve in organ development [30]. *AtGAT1* encoded high affinity γ-aminobutyric acid (GABA) transporter and was highly expressed in flowers and under the condition of high GABA concentrations such as wounding and senescence [31]. *AtANT1* was specifially expressed in flowers and cauline leaves, and transported aromatic and neutral amino acids as well as arginine and indole-3-acetic acid [32]. AtAUX1 was known as an auxin influx carrier and mediated the import of IAA which was a tryptophan-like signaling molecular [33]. AtAUX1 regulated root gravitropism and promoted lateral root formation by facilitating IAA distribution between sinks and source tissues in the *Arabidopsis* seedling [34], [35]. AtLAX3, a homologue of AtAUX1, was likely to influence the rate of lateral root emergence by regulating the auxin inducible expression of cell-wall-remodeling genes [36], [37]. In the wild cherry *Prunus avium*, PaLAX1 promoted the uptake of auxin into cells and affected the content and distribution of free endogenous auxin [38]. In the AtCAT subfamily, nine members were identified based on their sequence similarity. AtCAT5 functioned as a high-affinity, basic amino acid transporter in an amino acid transport in yeast [39]. AtCAT3, AtCAT6 and AtCAT8 preferentially transported neutral or acidic amino acids [40]. Meanwhile, AtBAT1, similar to a yeast GABA transporter (UGA4), was isolated as a bi-directional amino acid transporter [41]. Recently, this gene was characterized to be expressed in the mitochondrial membrane and mediate the transport of GABA from the cytosol into mitochondria [42].

Although the roles of many AATs were revealed in extensive studies of *Arabidopsis*, none of OsAATs was functionally characterized. Therefore, there is an urgent need for a thorough bioinformatics analysis of the *OsAAT* gene family. This study will provide comprehensive analyses of *OsAATs*, including the identification of all *OsAAT* family members, the phylogenetic relationships between OsAATs and AtAATs, as well as their expression profiling in different organs/tissues and under the treatments of abiotic stresses. These results will to a great extent benefit functional validation of the rice *AAT* genes and broaden our understandings of the roles of plant AATs.

## Results

### Identification of *AAT* Genes in Rice Genome

To explore the entire gene family of *AATs* in rice, through two domain searches (PF01490 and PF00324) at MSU-RGAP (MSU-Rice Genome Annotation Project) database (http://rice.plantbiology.msu.edu/analyses_search_domain.shtml), we obtained 84 and 47 sequences, respectively; while by the InterPro of European Bioinformatics Institute database, 308 protein sequences of OsAATs from *japonica* were deposited. In the Rice Genome Express Database, using keyword searches of “amino acid transporter” and “amino acid permease”, 45 and 31 genes were identified, respectively. Sequences from these hits were used as queries employing BLAST algorithms to search against rice genome in the databases of both MSU-RGAP and NCBI. By removal of the sequence redundancies and alternative splice forms of the same gene, we initially identified 87 putative *AAT* genes in rice. All candidate AAT sequences were subjected to InterProScan for searching the presences of AAT domains. Four candidates were excluded from further analysis because they only contained short and incomplete AAT domains. Taken together, a total of 85 *AATs* were identified in rice. Meanwhile, similar methods were used to identify family members of *AATs* in *Arabidopsis*, and a total of 63 *AtAATs* were subsequently discovered. The detailed information about gene locus, FL-cDNA, ORF length for each *OsAAT* genes and characteristics of corresponding proteins are listed in Table 1, and those of *AtAATs* are showed in Table S1.

**Table 1:** The general information and sequence characterization of 85 *OsAAT* genes.

S.N.	Gene^a^	Accession Number		ORF^d^ (bp)	Protein^e^			TM^f^	Expression^g^
		RGAP Locus^b^	KOME^c^		Size (aa)	MW(D)	pI		
	AAP group								
1	*OsAAP1*	LOC_Os07g04180	AK120257	1464	487	52864	8.66	9	A B C D
2	*OsAAP2*	LOC_ Os06g12330	AK106762	1455	484	51898.8	8.32	11	A B C D
3	*OsAAP3*	LOC_Os06g36180	AK102763	1464	487	52783.8	7.93	11	A B C D
4	*OsAAP4*	LOC_Os12g09300	AK069508	1407	468	50891.2	8.27	9	A B C D
5	*OsAAP5*	LOC_Os01g65660	AK073884	1398	465	50002.2	8.77	11	A B C D
6	*OsAAP6*	LOC_Os01g65670	AK121636	1401	466	50085.5	8.68	11	A B C D
7	*OsAAP7*	LOC_Os05g34980	AK065891	1491	496	53513.2	8.02	9	A B C D
8	*OsAAP8*	LOC_Os01g66010	AK059325	1467	488	52871.4	8.28	9	A B C D
9	*OsAAP9*	LOC_Os02g01210	AK060685	1557	518	57206.4	8.63	9	A B C D
10	*OsAAP10*	LOC_Os02g49060	AK070030	1410	469	50249.8	8.03	10	A B C D
11	*OsAAP11*	LOC_Os11g09020	AK068003	1431	476	51346.8	8.77	9	A B C D
12	*OsAAP12*	LOC_Os12g09320	NA	1407	468	50067.7	8.04	9	C D
13	*OsAAP13*	LOC_Os04g39489	AK071044	1401	466	50746.9	8.15	9	A B C D
14	*OsAAP14*	LOC_Os04g56470	NA	1410	469	51377.8	8.64	9	B C D
15	*OsAAP15*	LOC_Os12g08130	AK061470	1428	475	51063.2	8.73	9	A B C D
16	*OsAAP16*	LOC_Os12g08090	AK061448	1428	475	51091.3	8.73	9	A B C D
17	*OsAAP17*	LOC_Os06g12350	AK106814	1524	507	53441.6	9.55	9	A B C D
18	*OsAAP18*	LOC_Os06g36210	AK071510	1425	474	50720.6	8.30	6	A B C D
19	*OsAAP19*	LOC_Os04g41350	NA	1236	411	43154.1	8.47	7	C D
	LHT group								
20	*OsLHT1*	LOC_Os08g03350	AK102015	1344	447	49830.8	9.45	10	A B C D
21	*OsLHT2*	LOC_Os12g14100	AK070297	1341	446	48941.5	8.77	11	A B C
22	*OsLHT3*	LOC_Os05g14820	NA	1371	456	49939.7	9.12	11	C D
23	*OsLHT4*	LOC_Os04g38860	NA	1335	444	47795.9	9.00	7	B C D
24	*OsLHT5*	LOC_Os04g47420	AK060598	1539	512	55030.7	9.11	11	A B C D
25	*OsLHT6*	LOC_Os12g30040	AK100852	1527	508	54950.1	8.97	11	A B C D
	GAT group								
26	*OsGAT1*	LOC_Os05g50920	AK106883	1446	481	51041.1	9.43	10	A B C D
27	*OsGAT2*	LOC_Os01g43320	NA	1365	454	48525.1	9.02	11	B C D
28	*OsGAT3*	LOC_Os01g63854	AK103684	1374	457	48769.2	8.73	9	A B C D
29	*OsGAT4*	LOC_Os10g27980	AK119782	1329	442	47984.5	8.92	10	A B C D
	ProT group								
30	*OsProT1*	LOC_Os01g68050	NA	1344	447	49024.9	9.40	10	B C D
31	*OsProT2*	LOC_Os03g44230	AK067118	1422	473	52053.6	7.90	11	A B C D
32	*OsProT3*	LOC_Os07g01090	AK066298	1305	434	47663.8	9.51	11	A B C D
	AUX group								
33	*OsAUX1*	LOC_Os01g63770	AK100090	1479	492	54762	8.15	10	A B C D
34	*OsAUX2*	LOC_Os05g37470	AK111849	1512	503	55662.9	8.65	11	A B C D
35	*OsAUX3*	LOC_Os03g14080	AK103524	1575	524	58083.4	9.21	10	A B C D
36	*OsAUX4*	LOC_Os10g05690	AK102295	1644	547	59846.2	8.64	10	A B C D
37	*OsAUX5*	LOC_Os11g06820	NA	1443	480	52956.5	9.33	9	B C D
	ANT group								
38	*OsANT1*	LOC_Os07g12770	AK105808	1275	424	45712.3	7.01	11	A B C D
39	*OsANT2*	LOC_Os03g60260	AK121940	1257	418	43571.8	7.79	9	A B C D
40	*OsANT3*	LOC_Os02g44980	AK100650	1269	422	44846.9	7.53	11	A B C D
41	*OsANT4*	LOC_Os04g47780	AK058888	1278	425	44788.8	7.84	9	A B C D
	ATLa group								
42	*OsATL1*	LOC_Os06g43700	AK070101	1461	486	51755.2	7.55	10	A B C D
43	*OsATL2*	LOC_Os09g26290	NA	927	308	32736.3	8.61	4	C D
44	*OsATL3*	LOC_Os02g49510	AK069154	1347	448	48108.2	7.18	11	A B C D
45	*OsATL4*	LOC_Os06g16420	AK120497	1347	448	48121.5	7.30	11	A B C D
46	*OsATL5*	LOC_Os06g42720	AK101315	1377	458	49977.6	6.42	10	A B C D
47	*OsATL6*	LOC_Os02g09810	AK069748	1380	459	50149.7	6.45	10	A B C D
48	*OsATL7*	LOC_Os01g61044	AK102220	1380	459	47870.2	9.80	11	A B C D
	ATLb group								
49	*OsATL8*	LOC_Os11g19240	NA	1452	483	50225.9	9.08	11	C D
50	*OsATL9*	LOC_Os02g54730	AK066747	1647	548	59332.3	5.07	10	A B C D
51	*OsATL10*	LOC_Os12g38570	AK069006	1785	594	63407.2	4.63	10	A B C D
52	*OsATL11*	LOC_Os02g01100	AK074023	1725	574	62911.4	5.92	9	A B C D
53	*OsATL12*	LOC_Os06g12320	AK107472	1242	413	43792.8	9.41	11	A B C D
54	*OsATL13*	LOC_Os04g38680	AK106202	1368	455	47994.6	8.30	10	A B C D
55	*OsATL14*	LOC_Os04g38660	NA	690	229	24225.6	9.39	6	C
56	*OsATL15*	LOC_Os01g41420	NA	1896	631	67357.3	9.70	10	B C D
57	*OsATL16*	LOC_Os01g41400	NA	1332	443	47386.9	8.75	10	C D
58	*OsATL17*	LOC_Os01g40410	NA	1383	460	49420.1	5.89	10	C
	CAT group								
59	*OsCAT1*	LOC_Os01g11160	AK099094	1851	616	65510.9	8.13	14	A B C D
61	*OsCAT2*	LOC_Os02g43860	AK101068	1818	605	64436.6	7.57	13	A B C D
60	*OsCAT3*	LOC_Os03g43970	NA	1329	442	46938	8.54	12	B
62	*OsCAT4*	LOC_Os03g45170	AK066436	1920	639	68202.9	5.74	14	A B C D
63	*OsCAT5*	LOC_Os04g45950	AK064289	1686	561	60780.4	8.3299	13	A B C D
64	*OsCAT6*	LOC_Os06g34830	AK063382	1791	596	61918.3	8.42	14	A B C D
65	*OsCAT7*	LOC_Os10g30090	AK100205	1869	622	66053.4	6.73	14	A B C D
66	*OsCAT8*	LOC_Os11g05690	NA	1413	470	49758.9	5.96	12	B D
67	*OsCAT9*	LOC_Os12g06060	NA	1608	535	56371.8	7.26	13	C D
68	*OsCAT10*	LOC_Os12g41890	NA	1806	601	62597.4	8.44	14	D
69	*OsCAT11*	LOC_Os12g42850	AK064822	1866	621	66108	5.80	14	A B C D
	ACT group								
70	*OsBAT1*	LOC_Os01g42234	AK071623	1599	532	57024.1	8.51	12	A B C D
71	*OsBAT2*	LOC_Os01g71700	NA	1089	521	38637.4	9.055	12	B C D
72	*OsBAT3*	LOC_Os01g71710	NA	1566	362	55915.2	8.8742	9	C D
73	*OsBAT4*	LOC_Os01g71720	AK065371	1578	525	55991.8	7.76	13	A B C D
74	*OsBAT5*	LOC_Os01g71740	NA	1554	517	55683.9	8.70	12	B C D
75	*OsBAT6*	LOC_Os01g71760	NA	1527	508	54464.5	9.00	12	C D
76	*OsBAT7*	LOC_Os04g35540	AK072850	1593	530	57764.3	8.08	11	A B C D
	PHS group								
77	*OsLAT1*	LOC_Os02g47210	AK068055	1695	564	60174.1	6.18	12	A B C D
78	*OsLAT2*	LOC_Os03g25840	NA	993	330	35288.6	8.98	3	D
79	*OsLAT3*	LOC_Os03g25869	AK072316	1644	547	59972.5	12.42	5	A B C D
80	*OsLAT4*	LOC_Os03g25920	AK107064	1500	499	51811.2	8.96	10	A B C D
81	*OsLAT5*	LOC_Os03g37984	AK071314	1653	550	60375	7.27	10	A B C D
82	*OsLAT6*	LOC_Os08g41370	NA	579	192	21285.3	8.97	5	/
83	*OsLAT7*	LOC_Os12g39080	AK099986	1344	447	48989.6	12.27	5	A B C D
84	*OsLAT8*	LOC_Os01g19850	NA	2391	796	88296.8	7.9546	7	B C D
85	*OsLAT9*	LOC_Os08g23440	NA	2970	989	108454	6.2146	11	B C D

a Systematic designation given to rice *AATs* in this study.

b Locus identity number of *OsAATs* assigned by RGAP.

c Full-length cDNA accession number of *OsAATs* obtained from KOME.

d Length of the open reading frame for *OsAATs.*

e Protein characterization of OsAATs obtained from RGAP.

f Number of transmembrane segments possessed by OsAATs, predicted by the TMHMM Server v2.0.

g Evidence for gene expression from (A) full-length cDNA, (B) ESTs, (C) microarray data, (D) massively parallel signature sequencing (MPSS).

S.N., serial number; ORF, open reading frame; bp, base pair; aa, amino acids; MW, molecular weight; pI, isoelectric point; TM, transmembrane; NA, not available.

The numbers and positions of exons and introns were determined through the comparison of full-length cDNA sequences and the corresponding genomic DNA sequences of each *OsAAT* gene by using GSDS (http://gsds.cbi.pku.edu.cn/). Introns are absent in nine coding sequences of *OsAAT* genes, and the number of introns in other coding sequences range from one to thirteen (Figure S1). In the same subfamily, most members share similar intron/exon structures and gene lengths. For example, the three members of the *OsProT* subfamily have six introns and seven exons, and are nearly 4000 bp in length. The putative transmembrane (TM) regions in OsAATs were predicted by TMHMM Server v2.0 (http://www.cbs.dtu.dk/services/TMHMM/). The number of TM regions in most OsAATs ranges from eight to thirteen (Figure S2), and OsAATs of the same subfamily have similar number of TM regions, such as 11 TMs in LHT, 14 TMs in CAT, and 13 TMs in ACT. These observations revealed that members of the same subfamily were very conservative in structure.

### Chromosomal Localization and Gene Duplication

**Figure 1:**
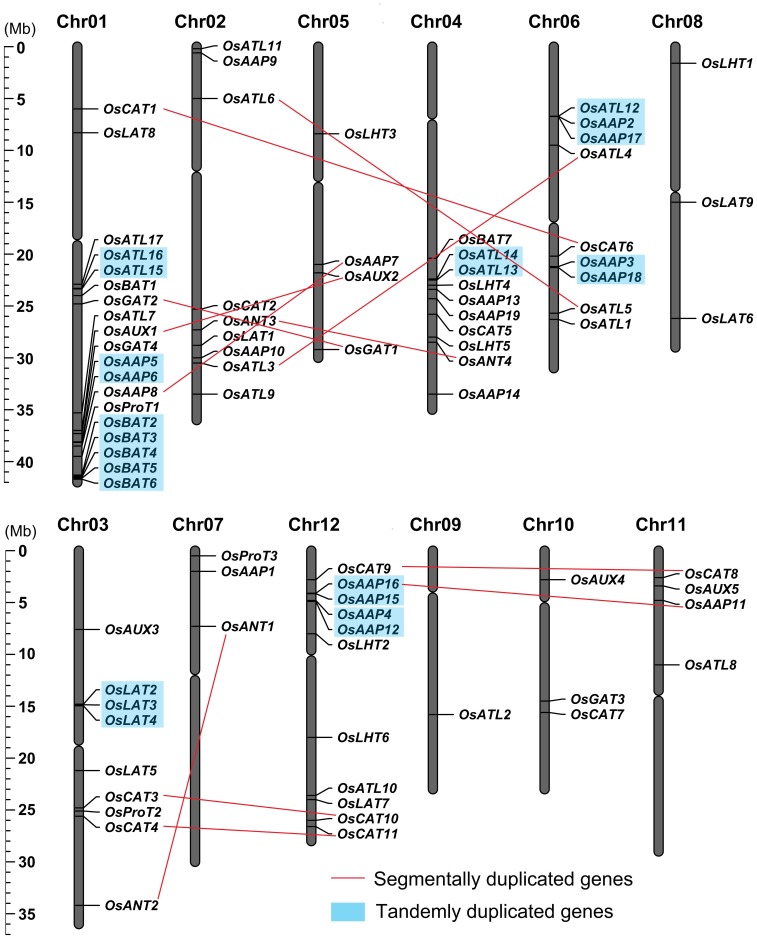
Chromosomal localization and gene duplication events of *OsAAT* genes. Respective chromosome numbers are indicated at the top of each bar. The scale on the left is in megabases (Mb). The cleavages on the chromosomes (vertical bars) indicate the position of centromeres. The chromosome order is arranged to bring duplicated regions in the vicinity.

To investigate the relationship between the genetic divergence and gene duplication within the *AAT* family in rice, the chromosomal locations of *OsAAT* genes were determined based on the coordinates of RGAP loci (http://rice.plantbiology.msu.edu/cgi-bin/gbrowse/rice/#search). *OsAAT* genes are distributed in all of rice 12 chromosomes. The densities of *OsAAT* genes are relatively higher in specific chromosomal regions, such as the bottom of chromosome 1 and 4, and the top and bottom of chromosome 2 and 12. In contrast, several large chromosomal regions are devoid of *OsAAT* genes, such as in the top parts of chromosomes 4 and 9, and in the bottom of chromosomes 7 and 11. The maximum members (19) of *OsAAT* genes are located in chromosome 1, followed by 11 genes in chromosome 12 and 10 genes in chromosome 4. In addition, nine genes each are located on chromosome 2, 3, and 6; four genes each on chromosome 5 and 11, three genes each on chromosome 7, 8, and 10. Nevertheless, a unique gene is localized on chromosome 9 (Figure 1). Similarly, the analysis of chromosomal location and gene duplication of 63 *AtAATs* was also performed in *Arabidopsis* (Figure S3).

Among the rice 85 *OsAAT* genes, 55.29% (47 of 85) come from the duplication events, including 24 gene segmental duplication and 23 gene tandem duplication. The 24 (12 pairs) *OsAAT* genes could be assigned to rice segmental duplication blocks based upon the analysis of the MSU-RGAP segmental duplication database. Four pairs of *OsCATs*, two pairs each of *OsAAPs*, *OsANTs* and *OsATLs*, and one pair each of *OsGATs* and *OsAUXs* are located on the duplicated segmental regions of rice chromosomes mapped by TIGR when the maximal length between collinear gene pairs is 500 kb. All of these duplicated genes exhibit high sequence similarity in both transmembrane regions and other domains. According to the criterion of separation by less than 5 intervening genes and ≥50% similarity at protein level, a total of 23 genes were found to be tandemly duplicated, falling into nine groups. Six groups comprised 2 genes and two groups comprised 3 genes in each group, and one group comprised 5 genes. The tandemly duplicated genes are only localized in five chromosomes, 3 groups on chromosome 1, 2 groups each on chromosome 6 and 12, and one group each on chromosome 3 and 4 (Figure 1). Moreover, all of the proteins of the duplicated genes have relatively high sequence similarity. For example, OsAUX1 and OsAUX2 from segmental duplication are 82% similarity, and OsAAP3 and OsAAP18 from tandem duplication are 70% similarity. These results suggested that large-scale segmental and tandem duplication events played a significant role in the expansion of the *OsAAT* family.

### Phylogenetic Analysis and Multiple Sequence Alignment

**Figure 2:**
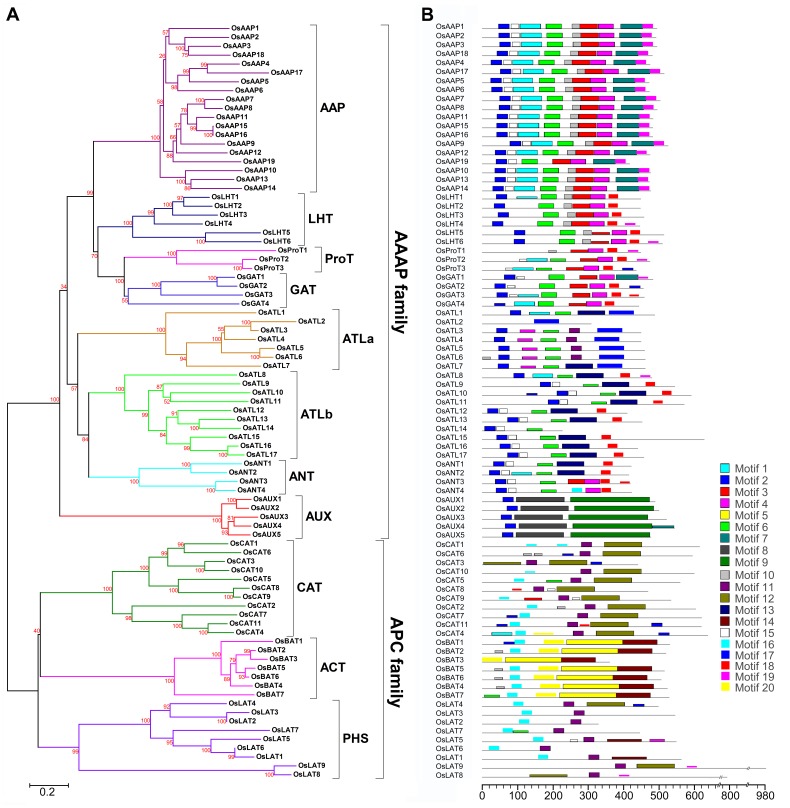
Phylogenetic relationship and protein motifs of OsAATs. (A) Phylogenetic tree of OsAATs constructed by neighbor-joining method. Bootstrap values from 1000 replicates are indicated at each node. Scale bar represents 0.2 amino acid substitution per site. The proteins on the tree can be divided into eleven distinct subfamilies. Subfamilies ATL are further divided into two groups (ATL a and ATL b). The branches of different subfamilies are marked by different colors. (B) Protein motifs of OsAATs. Each colored box represents a specific motif in the protein identified using the MEME motif search tool. The order of the motifs corresponds to their position within individual protein sequences.

In order to evaluate the evolutionary relationship among the 85 members of OsAATs, phylogenetic analysis was performed based on the alignment of full-length amino acid sequences of the 85 AAT proteins. Eleven distinct clades supported with high bootstrap values by the neighbor-joining method were identified. Additionally, baysian inference was also used to construct the phylogeny by using MrBayes program (Figure S4). Based on the domain composition and phylogenetic relationship, the OsAATs can be divided into two main families similar to that in *Arabidopsis*, including the AAAP and APC family (Figure 2A). The AAAP family consists of 58 OsAATs, including eight distinct subfamilies: amino acid permeases (AAPs), lysine, histidine transporters (LHTs), proline transporters (ProTs), GABA transporters (GATs), auxin transporters (AUXs), aromatic and neutral amino acid transporters (ANTs) and amino acid transporter-like (ATL) subfamilies. So far, the characterization of these genes in the ATL subfamilies has not been reported in *Arabidopsis* and other plant species. ATL subfamilies consist of two phylogenetic clades that are named as ATLa and ATLb, respectively. The five auxin transporters in OsAUXs subfamily were initially described as auxin influx-like carriers (OsLAX1-5) [43], [44]. Since the abbreviation LAX in rice was also used for the lax panicle and *OsLAX1* and *OsLAX2* were functionally characterized to regulate the formation of axillary meristems [45], [46], the five auxin transporters in rice were renamed to OsAUX1-5. The APC family is comprised of 27 OsAATs and subdivided into three distinct subfamilies, including the cationic amino acid transporters (CATs), the amino acid/choline transporters (ACTs) and the polyamine H^+^-symporters (PHSs).

In order to identify orthologous genes between rice and *Arabidopsis*, a combined phylogenetic tree with OsAATs and AtAATs was also established by using the neighbor-joining method (Figure S5). Similar subfamilies were formed as compared to the tree of OsAATs. Each clade of distinct subfamily consists of AATs from both rice and *Arabidopsis*, indicating the main characteristics formation of AAT family in rice and *Arabidopsis* before the split of monocotyledonous and dicotyledonous plants. Moreover, the difference in the total number of *OsAATs* and *AtAAT*s is mainly due to the variation in the number of genes in *AAP* subfamily and APC family, there were 19 AAPs in rice and 8 in *Arabidopsis*; 27 APCs in rice and 15 in *Arabidopsis*.

Moreover, the MEME motif search tool was employed to identify the motifs shared in the OsAATs. Subsequently, 20 motif sequences were identified and shown in Table S2. Besides, the distributions of these motifs in OsAATs were shown in Figure 2B. Several motifs were widespread among OsAATs in AAAP family (e.g. motif 2 and 6). In contrast, other motifs were specific to only one or two subfamilies. For example, motifs 1 and 7 were specific to subfamily AAPs, and motifs 8 and 5 were specific to AUXs and ACTs, respectively, while motif 12 and 17 were found in subfamily CAT and ATLa, respectively. However, motif 15 was present in the AAP, GAT, ATLb and ANT subfamilies. In addition, motif 11 exclusively appeared in the ATLa, CAT and PHS subfamilies.

**Figure 3:**
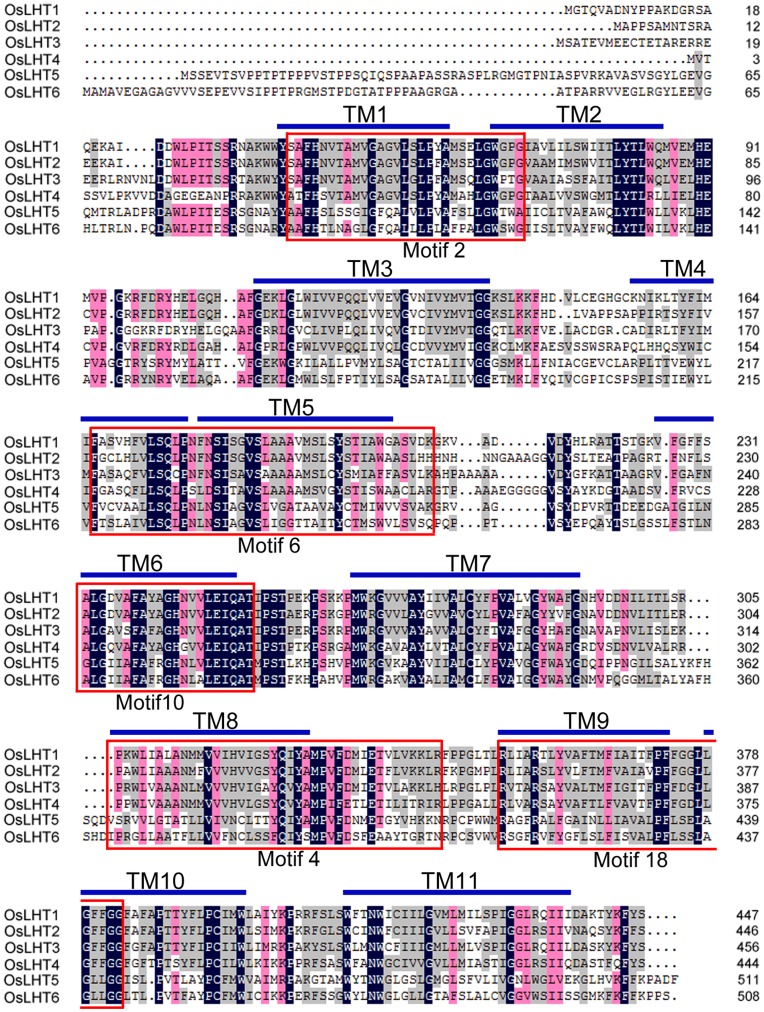
Multiple sequence alignment and transmembrane region of OsAATs. Identical (100%), conservative (75-99%) and block (50-74%) of similar amino acid residues are shaded in deep blue, cherry red and gray, respectively. The transmembrane regions are marked by blue lines. The conserved motifs 2, 6, 10, 4, 18 are orderly marked by red rectangles.

The alignments of the OsAATs’ amino acid sequences illustrated that most of TM regions in the same subfamily were very conserved. In addition, several TM regions of different members varied insignificantly both in length and amino acid composition. The alignment of the OsLHT members was shown in Figure 3 as an example. The overall identities of the protein sequences of these genes are 55.75%. There are five conserved motifs in OsLHTs, including motif 2, 6, 10, 4 and 18. Motif 2 was found to be located in the first and second TM region, and motif 6 comprised the fourth and fifth TM region. Motif 10 consisted in the sixth TM region. Motif 4 was located at the eighth TM region and extended into the following sequences before the ninth TM region. Motif 18 was located in the tenth and eleventh TM region.

### Expression Analysis of *OsAAT* Genes in Various Organs at Different Developmental Stages

Several approaches were employed to analyze the expression patterns of the *OsAAT* genes in different tissues and organs, including expressed sequence tag (EST) profiles, microarray data and massively parallel signature sequencing (MPSS) tags.

In analysis of EST profiles (http://www.ncbi.nlm.nih.gov/unigene/), the availability of any corresponding full-length cDNA (FL-cDNA) and ? or ESTs in UniGene database at NCBI was searched. Seventy-one of 85(83.53%) *OsAAT* genes have at least one corresponding FL-cDNA and ? or EST sequence (Table S3); fifty-nine *OsAAT* genes of them (69.41%) have both FL-cDNA and EST evidence, whereas twelve genes (14.12%) have only EST evidence (Table 1), indicating that most of these genes are expressed. Various *OsAAT* genes show high expression abundance in stem, root, leaf, panicle, and seed, several of which have tissue-specific or abundant expression patterns: *OsAAP6*, *OsAAP14*, and *OsAUX3* in callus; *OsLHT2*, *OsATL10* and *OsLAT5* in flower; *OsATL15*, *OsLAT7* and *OsCAT11* in leaf; *OsAAP1*, *OsAAP15* and *OsAAP17* in panicle; *OsLHT1*, *OsAUX1* and *OsBAT7* in root; *OsBAT5* in seed and *OsBAT4* in stem (Table S3).

**Figure 4:**
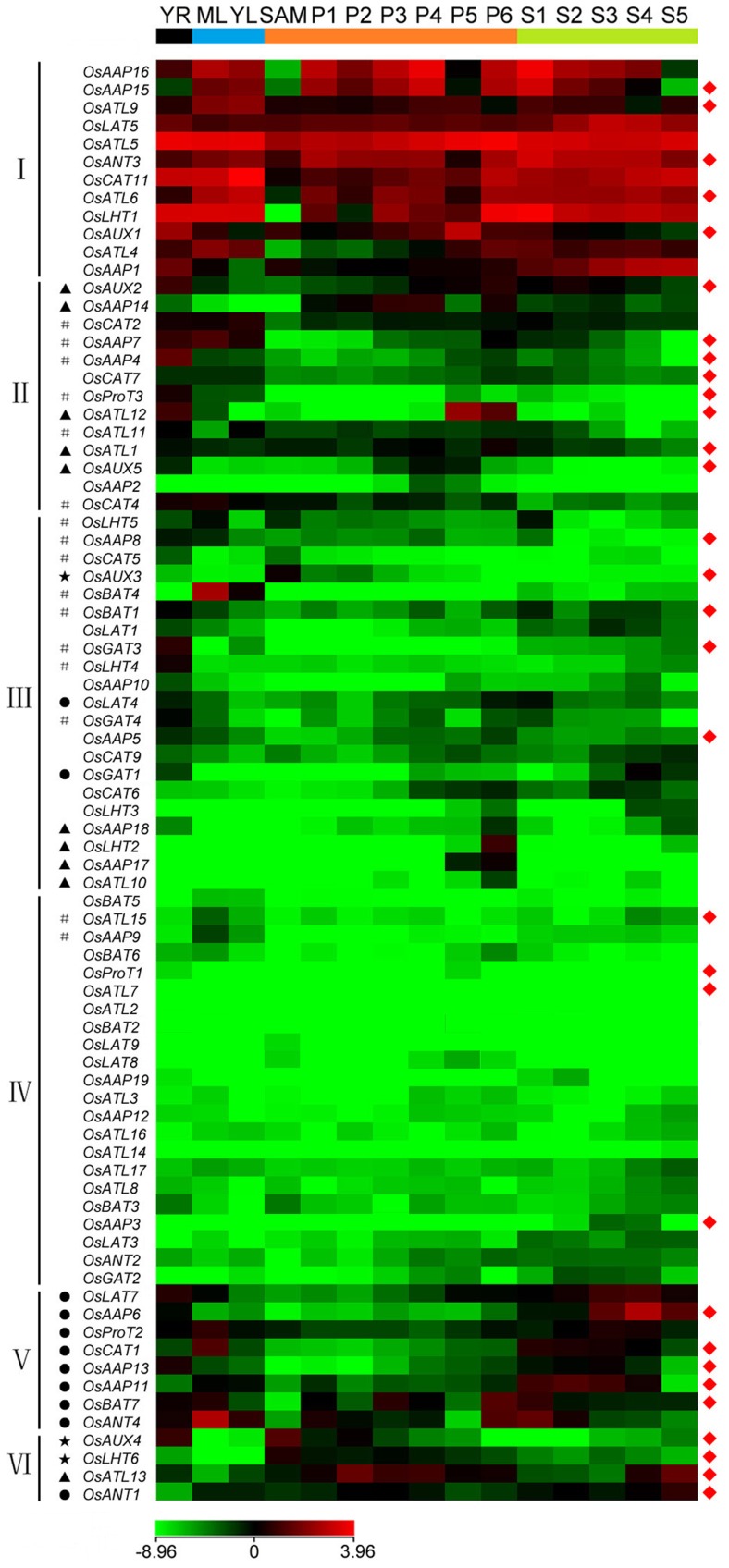
Expression profiles of *OsAAT* genes in various organs. The microarray data sets (GSE6893) of *OsAAT* genes expression in organs at various developmental stages were reanalyzed. A heat map representing hierarchical cluster in various organs are generated. Color key represents average log2 expression values of *OsAAT* genes. Samples are indicated at the top of each lane: YR, roots from 7-day-old seedlings; ML, mature leaf; YL, leaves from 7-day-old seedling; SAM, shoot apical meristem; different stages of panicle development: P1, 0-3 cm; P2, 3-5 cm; P3, 5-10 cm; P4, 10-15 cm; P5, 15-22 cm; P6, 22-30 cm; different stages of seed development: S1, 0-2 dap (day after pollination); S2, 3-4 dap; S3, 5-10 dap; S4, 11-20 dap; S5, 21-29 dap. Genes that share similar expression patterns are divided into six groups (?-?). Asterisks (*), hash symbols (#), triangles (▴) and rounds (•) indicate the genes with preferential expression level in SAM, YR and/or ML/YL, P1-P6 and S1-S5, respectively. The representative *OsAAT* genes differentially expressed in various organs for which real-time PCR analysis was performed are indicated by a red diamond (?) on the right. The colour scale (representing average log signal values) is shown at the bottom.

Microarray data (http://signal.salk.edu/cgi-bin/RiceGE) of various tissues during vegetative and reproductive developmental stages of rice was used for analyzing the expression profiles of *OsAAT* genes, including young root (YR), mature leaf (ML), young leaf (YL), shoot apical meristem (SAM), panicles (P1–P6), and seed (S1–S5) development. At least one probe could be found on Affymetrix rice whole-genome array platform (GPL2025) for 80 of 85 *OsAAT* genes. A hierarchical cluster display generated from the average log signal values for the 80 *OsAAT* genes in selected organs indicates the differential expression patterns of these genes (Table S4). The expression patterns of 80 *OsAAT* genes can be divided into six major groups (Figure 4). Twelve *OsAAT* genes in group I show high expression levels in all examined organs, expect for five genes with relatively low expression in certain organs (*OsAAP16* and *OsLHT1* in SAM, *OsAAP15* in SAM and S5, *OsATL4* in SAM and P1–P3, and *OsAAP1* in YL). Group II includes 13 genes that show abundant expression level in vegetative organs. For example, four genes (*OsAUX2*, *OsAAP4*, *OsProT3* and *OsATL12*) display specifically or predominantly expression level in YR; *OsCAT2*, *OsAAP7* and *OsCAT4* were abundantly expressed in YR, ML and YL. Group III comprises 21 genes that show relatively high expression level in specific organs (*OsAUX3* in SAM, *OsBAT4* in YL and ML, *OsBAT1*, *OsGAT3*, *OsGAT4* and *OsLHT4* in YR, *OsCAT6*, *OsAAP18*, *OsLHT2*, *OsAAP17* and *OsATL10* in P6). Conversely, group IV contains 18 genes that show relatively low expression level in all examined organs. Group ? comprises 8 genes with preferential expression patterns in different stages of seed development, meanwhile, *OsProT2* and *OsCAT1* are highly expressed in ML, and *OsAAP13* and *OsLAT7* in YR. Group VI consists of 4 genes which show relatively high expression levels in several certain reproductive organs (*OsAUX4* and *OsLHT6* in SAM, *OsATL13* in P1–P4 and S4–S5, *OsANT1* in S4–S5).

In the mRNA MPSS database of rice (http://mpss.udel.edu/rice/), the expression of *OsAAT* genes were investigated. MPSS generates hundreds of thousands of molecules per reaction and provides a quantitative assessment of transcript abundance. MPSS signatures are available for the 80 genes in at least one of the libraries (Table S5), further indicating that most *OsAAT* genes are expressed. Differential expression abundances are displayed by number of tags (tpm, transcripts per million), being low in <50, moderate in between 50 tpm and 500 tpm, and strong in >500 tpm. A large percentage of *OsAAT* genes (47 of 80) have low expression level, 30 genes moderate level, and three genes (*OsAUX1*, *OsATL5* and *OsATL15*) high level (Table S5).

**Figure 5:**
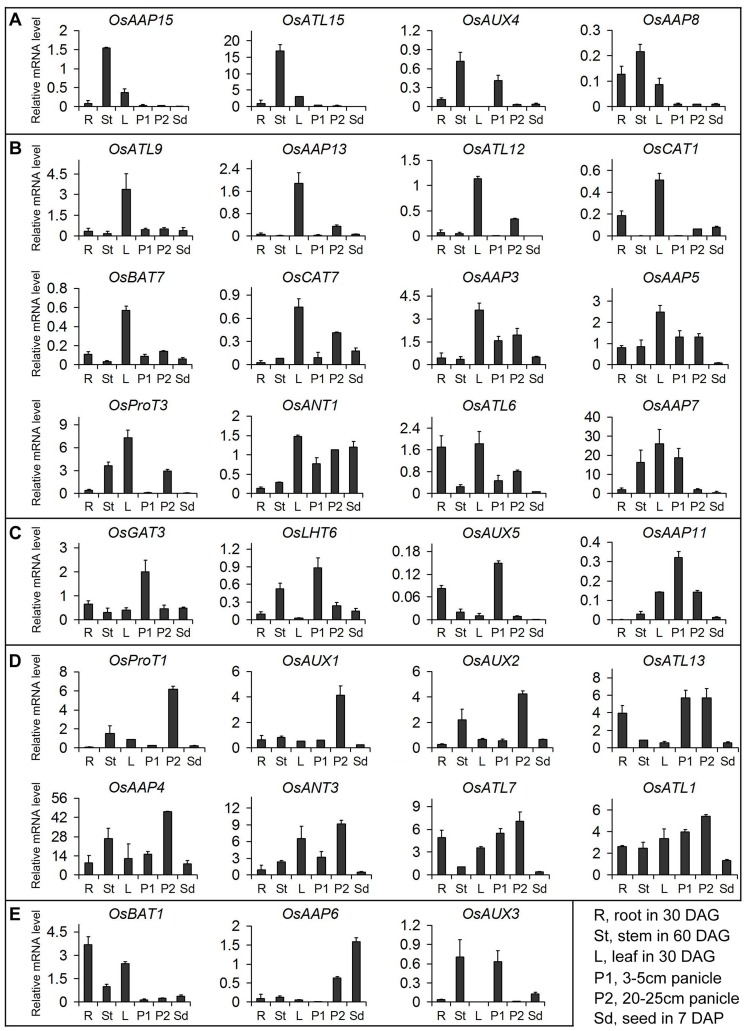
Real-time PCR analysis of tissue-specific expression of the representative *OsAAT* genes. Relative mRNA levels of individual genes normalized to *UBQ5* are shown. Y-axis shows the relative mRNA expression level; X-axis shows different organs. (A), (B), (C) and (D) Showing the genes with preferential expression in St, L, P1 and P2, respectively. (E) Showing the genes with preferential expression in R, Sd, St and P1. Error bars indicate standard deviations of independent biological replicates (n  =  2 or more).

To validate the results of digital expression analysis, we detected the expression levels of several representative genes by real-time PCR analysis. The results show that they are in general agreement with the data of microarray and MPSS tags. For example, *OsAAP15*, *OsATL15*, *OsAUX4* and *OsAAP8* are significantly expressed in stems, meanwhile, *OsAAP15*, *OsATL15* and *OsAAP8* are also expressed in roots and leaves, and *OsAUX4* in root and in panicles at early stages of development (P1) (Figure 5A). *OsATL9*, *OsCAT1*, *OsBAT7*, *OsAAP5*, *OsANT1*, *OsATL6* and *OsAAP7* are predominantly or highly expressed in leaves (Figure 5B). *OsLHT6*, *OsAUX5* and *OsAAP11* are mainly expressed in panicles at early stages of development (P1), and *OsAUX5* is also highly expressed in roots (Figure 5C). Otherwise, *OsProT1*, *OsAUX1*, *OsAUX2*, *OsATL13*, *OsAAP4*, *OsANT3* and *OsATL1* are predominantly or highly expressed in panicles at late stages of development (P2), and *OsATL13* similarly in panicles at early stages of development (Figure 5D). *OsBAT1* and *OsAAP6* are highly expressed in roots and seeds, respectively. *OsAUX3* is predominantly expressed in stem and P1, but was not detected in either leaf or P2 (Figure 5E).

### Expression Regulation of *OsAAT* Genes under Abiotic Stresses

**Figure 6:**
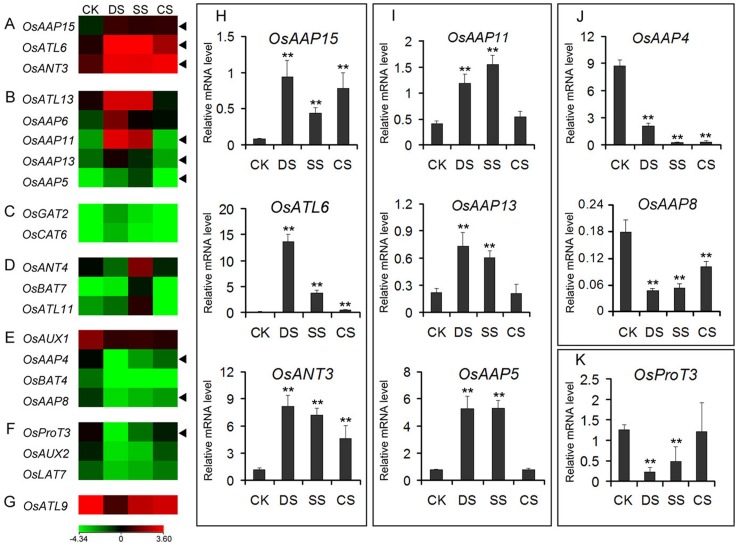
Differential expression profiles of *OsAAT* genes under abiotic stresses. The microarray data sets (GSE6901) of gene expression under various abiotic stresses were used for cluster display. The average log signal values of *OsAAT* genes under control and various stress conditions (indicated at the top of each lane) are presented by a heat map. Only those genes that exhibited >2-fold or more differential expression, under any of the given abiotic stress conditions, are shown. The color scale representing average log signal values is shown at the bottom. The representative *OsAAT* genes differentially expressed under different abiotic stresses for which real-time PCR analysis was performed are indicated by black triangle at the right. The mRNA levels for each candidate gene in different samples were calculated relative to its expression in control seedlings. Error bars indicate standard deviations of independent biological replicates (n  =  3 or more). Two asterisks (**, *P* <0.01, Student’s *t*-test) represent significant differences between the controls and treatments. CK, control; DS, drought stress; SS, salt stress; CS, cold stress.

To determine the response of *OsAAT* genes to abiotic stress, the data of microarray (GSE6901) of 7-day-old seedlings subjected to the treatments of drought, salt and cold stresses were analyzed. It was revealed that a total of 21 genes were evidently down- or up-regulated (<0.5 or >2) in at least one of the stress conditions examined as compared with the control (Figure 6A–G). The expression of three genes (*OsAAP15*, *OsATL6* and *OsANT3*) were up-regulated by all three stresses (Figure 6A), five genes (*OsATL13*, *OsAAP6*, *OsAAP11*, *OsAAP13* and *OsAAP5*) were up-regulated by drought and salt stresses (Figure 6B), and two genes (*OsGAT2* and *OsCAT6*) and three genes (*OsANT4*, *OsBAT7* and *OsATL11*) were specifically up-regulated by drought and salt stress, respectively (Figure 6C and D). However, four genes (*OsAUX1*, *OsAAP4*, *OsBAT4* and *OsAAP8*) were down-regulated by all three stresses (Figure 6E), three genes (*OsProT3*, *OsAUX2* and *OsLAT7*) were down-regulated by drought and salt stresses (Figure 6F), and one gene (*OsATL9*) was down -regulated by drought stress (Figure 6G). By using real-time PCR analysis, we validated the expression levels of 9 representative *OsAAT* genes in 7-day-old seedling under three stress conditions. It was found that the results were in very well agreement with the microarray data (Figure 6 H, I, J and K), indicating that *OsAAT* genes might participate in abiotic stress signaling pathways and play important roles in response to these stresses.

### Comparative Expression Analysis of Rice and *Arabidopsis AAT* Genes

In order to explore valuable clues for the study of gene function, a comparative analysis of the expression patterns of rice and *Arabidopsis AAT* gene family was performed. With the exception of the expression data of *OsAAT* genes in pollens from MPSS tags, the other expression data of rice and *Arabidopsis AAT* genes were extracted from microarrays in root, leaf, inflorescence, pollen, seed/silique, and under abiotic stress. The ratios of the absolute values divided by the average of all microarray values were used for the analysis (Table S6).

**Figure 7:**
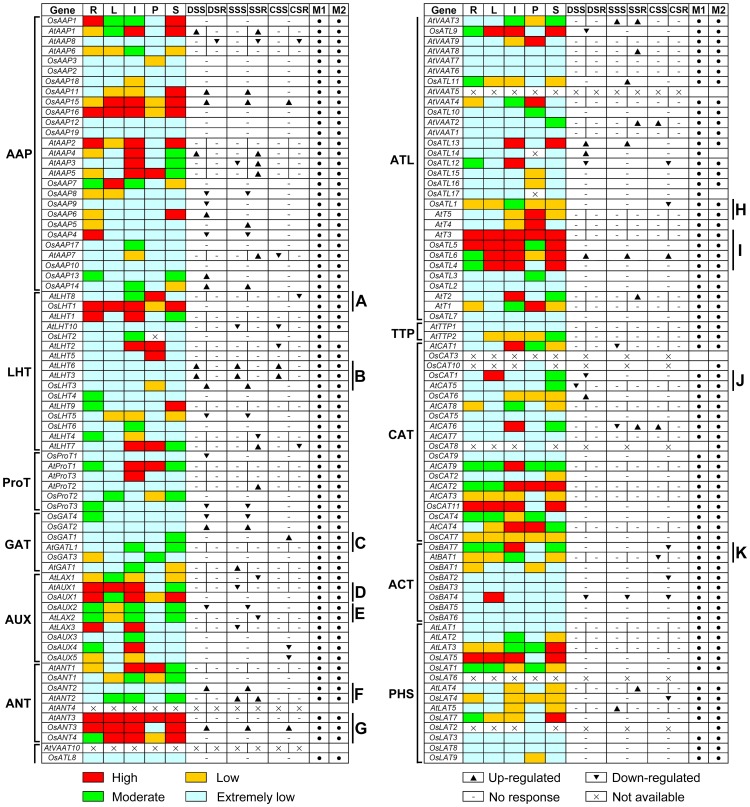
Expression comparison between rice and *Arabidopsis AAT* genes in different organs and under abiotic stresses. The *OsAAT* and *AtAAT* genes are displayed according to the order in the corresponding phylogenetic tree (Figure S5). The expression data of *OsAAT* genes in different organs are combined from microarrays (M1) and MPSS (M2). In microarrays and MPSS data, red, green, yellow and light blue boxes indicate high (more than 2, 300 tpm), moderate (between 1 and 2, between 50 and 300 tpm), low (between 0.5 and 1, the signature numbers between 0 and 50 tpm), and extremely low (less than 0.5, no signature) expression levels, respectively. The symbol “×” represents no probe or signature on microarray and MPSS. “▴”, “▾” and “-” represent expression values that are evidently higher(>2), lower(<0.5) and no evident difference(0.5-2) under abiotic stresses compared to the control, respectively. (A) and (B-K) Showing homologous genes with distinct and similar expression patterns, respectively. R, root; L, leaf; I, inflorescence; P, pollen; S, silique (*Arabidopsis*) or seed (Rice); DSS and DSR; drought stressed shoot and root; SSS and SSR, salt stressed shoot and root; CSS and CSR, cold stressed shoot and root.

Most of the rice and *Arabidopsis AAT* genes were found to be presented on at least one data set, while five genes (*OsCAT3*, *OsLAT6*, *AtANT4*, *AtVAAT10* and *AtVAAT5*) were absent from the two data sets (Figure 7). By integrating the data of microarray and MPSS tags, we found that 28 *AAT* genes were highly expressed in at least two organs examined and 21 *AAT* genes acted in tissue-specific manners. The expression levels of 36 *AAT* genes were extremely low in all examined organs. Six members of GAT subfamily and 4 of 6 in ProT subfamily were moderate and low expressed in all examined organs. On the contrary, nine members of AUX subfamily were high and/or moderate expressed in at least one organ except *OsAUX5* that were lowly expressed in all examined organs.

Meanwhile, this comparison also indicated that 26 *AAT* genes with close evolutionary relationships between rice and *Arabidopsis* showed similar expression patterns. Correlation coefficients between expression patterns ranged from 0.7 to 0.9 among most homologous genes (Table S7). For example, *AtLHT6*, *AtLHT3* and *OsLHT3* were very lowly expressed in all organs examined and evidently up-regulated in stems by drought and salt stresses (Figure 7B). *OsAUX2* and *AtLAX2* were mainly expressed in root, inflorescences and seeds and evidently down-regulated by salt stress (Figure 7E). *OsCAT1* and *AtCAT5* showed moderate or extremely low expression level except *OsCAT1* in leaf and were down-regulated by drought stress (Figure 7J). *OsANT2* and *AtANT2* were up-regulated by salt stress (Figure 7F), and OsBAT7 and AtBAT1 were down-regulated by cold stress (Figure 7K). Moreover, *OsGAT1* and *AtGATL1* were moderately expressed in seeds (Figure 7C), *OsAUX1* and *AtAUX1* had high or moderate expression level in root, leaf, inflorescences and seeds (Figure 7D). *OsANT3* and *AtANT3* were highly expressed in all organs except *OsANT3* in pollens (Figure 7G), while OsATL1 and AtT5 were very lowly expressed in root, leaf and seeds (Figure 7H). *AtT3*, *OsATL4*, *OsATL5* and *OsATL6* were predominantly expressed in leaf, inflorescences and seeds (Figure 7I).

In addition, the expression of *OsAAT* genes in rice differed from those of their *Arabidopsis* homologs. For example, *OsLHT1* was highly expressed in root, leaf, inflorescences and seeds, while *AtLHT8* was only highly expressed in pollens, and had extremely low expression level in root, leaf, and seeds (Figure 7A). In conclusion, the expression patterns of genes provide foundation for future functional studies of *AAT* genes in both rice and *Arabidopsis*.

### Expression Pattern Divergence of Paralogous *OsAAT* Genes Involved in Duplication

**Figure 8:**
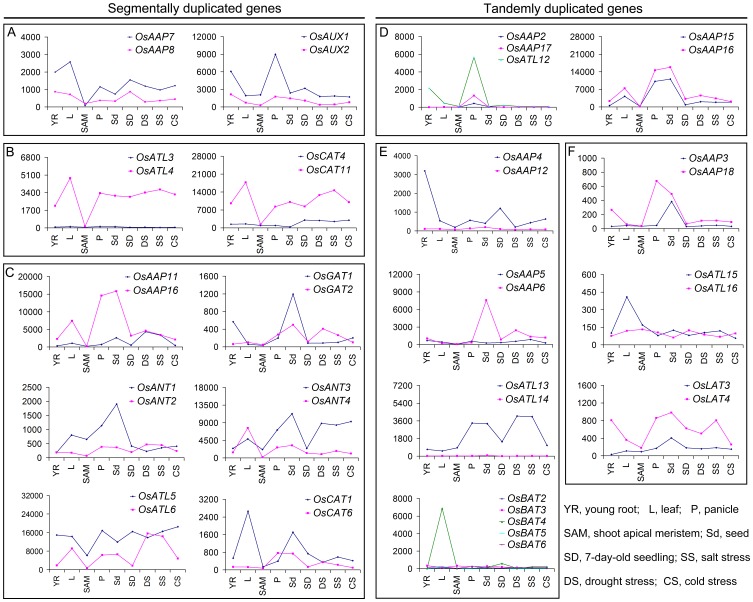
Expression divergence of paralogous *OsAAT* genes involved in duplication. The absolute values of duplicated genes obtained from microarray data were compared in various organs under abiotic stresses. X-axis indicates representative samples and Y-axis is scales of expression level. The segmentally and tandemly duplicated genes are shown on the left and the right, respectively. (A) and (D) Showing gene pairs that are retention of expression; (B) and (E) showing gene pairs described as non-functionalization; (C) and (F) showing gene pairs described as neo-functionalization.

The analysis on the expression pattern of *OsAAT* genes present in segment and in tandem duplication was performed. Out of the 12 pairs of segmentally duplicated genes, probe sets were available for 10 pairs on Affymetrix gene chip (http://signal.salk.edu/cgi-bin/RiceGE). The expression pattern was very much similar for 2 pairs of genes (*OsAAP7* and *OsAAP8*, *OsAUX1* and *OsAUX2*), indicating they were just formed due to duplication and retention of function (Figure 8A). The fate of 2 pairs (*OsATL3* and *OsATL4*, *OsCAT4* and *OsCAT11*) in Figure 8B could be described as nonfunctionalization, where one copy of the paralog had almost negligible expression in all organs, and which may be due to the fact that gene with low expression level would tend to lose its function in due course of evolution [47]. For the rest six pairs of paralogous genes (*OsAAP11* and *OsAAP16*, *OsGAT1* and *OsGAT2*, *OsANT1* and *OsANT2*, *OsANT3* and *OsANT4*, *OsATL5* and *OsATL6*, and *OsCAT1* and *OsCAT6*), the expression patterns were very divergent for one or more of the tissues detected, indicating neo-functionalization (Figure 8C).

Expression pattern analysis was also done for tandemly duplicated *OsAAT* genes. Two groups of genes, (*OsAAP2*, *OsAAP17* and *OsATL12*, *OsAAP15* and *OsAAP16*) had highly similar expression pattern and hence retention of expression (Figure 8D), whereas, for four groups of genes (*OsAAP4* and *OsAAP12*, *OsAAP5* and *OsAAP6*, *OsATL13* and *OsATL14*, *OsBAT2*, *OsBAT3*, *OsBAT4*, *OsBAT5* and *OsBAT6*), one copy of the paralog lost expression in all organs (Figure 8E). Furthermore, three pairs (*OsAAP3* and *OsAAP18*, *OsATL15* and *OsATL16*, *OsLAT3* and *OsLAT4*) showed divergent expression profiles (Figure 8F).

## Discussion

### Organization of *AAT* Family Genes in Rice

In previous reports, several *AAT* genes in *Arabidopsis* were classified and functionally characterized in detail, such as genes in the *AtAAP* subfamily [11], [12], [15], the *AtAUX* subfamily [33]–[37] and the *AtCAT* subfamily [39]–[42]. However, members of *AAT* family in rice are still unknown so far, and none of them is functionally characterized. In this study, we identified 85 *OsAAT* genes which were divided into nine subfamilies based on sequence similarity in amino acid, and there was obvious difference in numbers among subfamilies. The largest number found in *OsAAP* subfamily was nineteen, and the smallest found in *OsProT* subfamily was only three (Table 1). Combination phylogenetic analysis indicated that the rice *AAT* subfamilies were very good agreement with that in *Arabidopsis* (Figure S5), which revealed that *AAT* families in rice and *Arabidopsis* were formed before the split of monocotyledonous and dicotyledonous plants [4]. With the exception of *ATL*a and ATLb subfamilies, at least one gene in other nine subfamilies was functionally identified in *Arabidopsis*. In addition, due to the significant increase in the number of AAP subfamily genes and APC family genes, the number of 85 *OsAAT* members was far greater as compared to the number of 63 in *Arabidopsis* (Table S1). The increase in the number of *OsAAT* members reflects that expansion and rearrangement of the genome may be successfully undergone in the *OsAAT* family, and these transporters increased in rice might play an important role in order to adapt to specific functions.

Chromosomal mapping of *OsAAT* family genes show their variable distribution on 12 rice chromosomes, but its most members are localized on chromosome 1, 4 and 12. Meanwhile, duplicated segments are mainly presented on chromosome 1, 3, 6 and 12, and nine gene groups in tandem duplication are localized only on five chromosomes (three, one, one, two and two groups on chromosome 1, 3, 4, 6 and 12, respectively) (Figure 1). Gene structure analysis reveals that most members in the same *OsAATs* subfamily are structurally conserved in the number of intron/exon and gene length (Figure S1), which indicates their close evolutionary relationship and the introduced classification of subfamilies.

### Duplication Contributed to the Expansion and Functional Divergence of *OsAATs* Family

Gene duplication is thought to be an important means of gene family expansion and functional diversity during evolution, which may occur through three major pathways: chromosomal segmental duplication, tandem duplication and retroposition [48]–[50]. In the present study, our analysis on gene duplication reveals that 47 of 85(55.30%) *OsAAT* genes are duplicated genes, 24 genes (28.24%) are involved in the segmental duplication, and 23 genes (27.06%) in tandem duplication, indicating that segmental and tandem duplications contribute almost equally to the expansion of the *OsAAT* gene family. Moreover, 16 genes in *OsAPC* family with 27 members are involved in duplication events, which may be largely responsible for the number difference of the member between *OsAPC* and *AtAPC* genes.

When gene duplication occurs, expression patterns and original functions of these genes may be retained [51]. Consistent with that, the comparison analysis for expression pattern of paralogous *OsAAT* genes involved in duplication indicates that *OsAAP7* and *OsAAP8*, and *OsAAP15* and *OsAAP16*, localized on segmental and tandem duplication, respectively, exhibited similar expression patterns in both development stages and abiotic stresses, indicating their overlapping functions (Figure 8A and 8D). However, it was well known that a great degree of expression and functional divergence might be present between two duplicated genes due to the intense selection pressure and the need for the diversification [49]. Most duplicated *OsAAT* genes exhibit distinct expression patterns and response to various abiotic stresses, such as *OsANT3* and *OsANT4*, *OsCAT1* and *OsCAT6* (segmentally duplicated genes) (Figure 8C); *OsAAP4* and *OsAAP12*, *OsLAT3* and *OsLAT4* (tandemly duplicated genes) (Figure 8E and 8F). These results suggested that chromosomal duplication events not only facilitated the expansion of the *OsAAT* family, and also lead to expression divergences between duplicate genes, further contributing to the establishment of gene functional diversity during their evolution.

### Expression Patterns Divergence and Putative Function of *OsAATs*

The analysis on temporal and spatial expression patterns of *OsAAT* genes may provide useful information for establishing their putative functions [52]–[54]. Our microarray analysis showed that the expression patterns of the 80 *OsAAT* genes could be divided into six major groups. Some preferential or tissue-specific expression *OsAAT* genes were also identified. Only three (*OsAUX3*, *OsAUX4* and *OsLHT6*) of the 80 genes were found to exhibit either preferential or tissue-specific expression in SAM, sixteen in YR and/or YL and/or ML. In addition, ten and eleven genes were preferentially or specifically expressed during different development stages of panicles and seeds, respectively. Interestingly, several genes were most highly expressed in several specific tissues, for example, *OsATL12* were preferentially expressed in YR, P5 and P6, and *OsCAT1* and *OsANT4* had high expression level in ML, S1 and S2.

In *Arabidopsis*, *AtAUX1* and *AtLAX3* were primarily expressed in root and promoted lateral root formation [55], [56]. Our data showed that *OsAUX1*, an ortholog of AtAUX1 and *AtLAX3*, had high expression levels in all the organs, especially in YR and P5, and *OsAUX2*, *OsAUX4* and *OsAUX5* were almost preferentially expressed in YR. Similar expression patterns suggest that these root preferentially or specifically expressed genes might play important roles in root formation and development. The results of some representative genes from real-time PCR showed that four genes (*OsAAP8*, *OsAAP15*, *OsATL15* and *OsAUX4*) are predominantly or specifically expressed in stems. It is known that *AtAAP2* may play critical role in the long-distance transport of amino acid [7], [13]; *AtAAP3* with mainly expression in root vascular tissue may be involved in amino acid uptake from phloem [57]; AtAAP6 might be responsible for amino acid uptake from xylem [58]. Therefore, combining their phylogenetic relationship and role of the *AtAAPs*, we infer that *OsAAP8* and *OsAAP15* might participate in the uptake and long-distance transport of amino acid.

Moreover, we also identified one gene (*OsAAP6*) that was preferentially expressed in early stages of seed development. *AtAAP8* had been demonstrated that may be involved in amino acid uptake into the endosperm and supplying the developing embryo with amino acids during early embryogenesis [17]. It may be conjectured that these *AAT* members might also play critical roles in nutrient transport during seed development.

### Roles of *OsAATs* in Response to Abiotic Stresses and in Reproductive Development

The expression profile of a gene can provide a valuable clue for its functional study [53]. Our expression analysis by using qRT-PCR and/or microarray data reveal that a subset of *OsAAT* genes from different subfamilies shows significant and differential expression pattern under three abiotic stresses (Figure 6 and 7). It is known that amino acid transport is highly regulated by environmental signals, such as light, low temperature, high salt and/or drought [59]. The expression of *AtAAP4* and *AtAAP6* were reported to be down-regulated by salt stress in *Arabidopsis* [60], as well as *McAAT2* in *M. crystallinum* [61]. On the contrary, *AtProT2* [60], *McAAT1* [61], and *HvProT* [62] were found to be strongly induced by salt stress. Similarly, in our investigation, seven genes (e.g. *OsAAP4*, *OsAAP8*, *OsBAT4*) and 11 genes (e.g. *OsAAP6*, *OsAAP11*, *OsANT3*) in *OsAAT* family were evidently down- and up-regulated, respectively, under salt and drought stresses (Figure 6). The investigation suggests that the *OsAAT* genes may play a critical role in abiotic stress signaling in rice.

Many evidences demonstrate that there is an interaction between developmental processes and stress responses, and some genes may be co-regulated by both environmental factors and developmental cues [63]. It was reported that a network of rice genes are associated with stress response and seed development [64]. By expressional analysis from microarray data and qRT-PCR, we also found that several *OsAAT* genes (such as *OsANT3*, *OsAAP6*, *OsAAP11*, *OsATL13* and *OsAUX1*), which were differentially expressed during at least one of the panicles and seed developmental stages, were significantly down- or up-regulated by one or more of the stress conditions. These genes may play important roles in plant growth and response to different abiotic stress conditions during reproductive development. This suggests that a number of *OsAAT* genes are likely to be involved in main developmental processes and stress responses. And their direct relationship requires further experimental validation.

### Conclusion

In conclusion, the results of this study display the genomic framework, classification, duplication manner and conserved motifs of the 85 OsAAT members, along with their expression profiles during different developmental stages as well as under abiotic stress treatments. These data will provide an insight into further understanding of functions of AAT members and their roles in rice growth and development. Our findings would be valuable in selecting candidate genes for functional validation studies of AAT members in rice. However, future research by adopting RNAi and overexpression strategies or insertion mutagenesis is required to explore the precise role of individual *OsAAT* gene.

## Materials and Methods

### Plant Materials and Abiotic Stress Treatment

To analyze the expression pattern of representative *OsAAT* genes, the rice seedlings of *Nipponbare* were grown under normal conditions in Wuhan University. The tissues and organs for expression pattern analysis were: (i) 30-day-old root (R, root) and leaf (L, leaf); (ii) 60-day-old stem(St); (iii) 3–5 cm panicles (P1); (iv) 20–25 cm panicles (P2); and (v) seed at 7 DAP (day after pollination) (Sd). For stress treatments, the 7-day-old seedlings were carefully transferred onto paper at 28°C as drought stress after which were air dried, placed in 400 mM NaCl solution at 28°C as salt stress, and kept in sterile water for 3h at 4°C as cold stress. Parallel control samples were prepared by keeping the seedlings in water for 3 h. All materials were collected and immediately frozen in liquid nitrogen, and stored in −80°C environment until RNA extraction.

### Database Screening and Identification of *OsAATs*

Several approaches were employed for the mining of all putative AAT members in the rice genome. Firstly, BLASTP searches of AAT domains (PF01490 and PF00324) search were performed on website of MSU-RGAP (http://rice. plantbiology. msu.edu/domain_search shtml). Secondly, protein sequences of putative OsAAT members were downloaded from Search Interpro (http://www.ebi.ac.uk/interpro/ISearch?query=PF01490 and PF00324). Thirdly, key words “amino acid transporter” and “amino acid permease” were used as queries to search against Rice Genome Express Database (http://signal.salk.edu/cgi-bin/RiceGE). Resulting protein sequences were then used as queries to perform two database searches against MSU-RGAP (http://rice.plantbiology.msu.edu/) and NCBI (http://www.ncbi.nlm.nih.gov/). After removing the redundant sequences, the remaining protein sequences were submitted to InterProScan (http://www.ebi.ac.uk/Tools/InterProScan/) to confirm the existence of AAT domain. Information about full-length cDNA accessions, coding sequence length, gene structure for each gene and characteristics of proteins were obtained from KOME (Knowledge-based Oryza Molecular biological Encyclopedia) and MSU-RGAP. Gene structures of *OsAATs* were analyzed on the website of GSDS (Gene Structure Display Server) (http://gsds.cbi.pku.edu.cn/) [65]. To predict the putative TM regions in each OsAAT protein, the TMHMM Server v2.0 (http://www.cbs.dtu.dk/services/TMHMM/) was applied with default settings.

### Chromosomal Localization and Gene Duplication

*OsAAT* genes were mapped onto the corresponding rice chromosomes by identifying their chromosomal positions given in the RGAP (http://rice.plantbiology.msu.edu/analyses_search_ locus.shtml). By using the MapChart software [66], the distribution of *OsAAT* genes on chromosomes was drawn and modified manually with annotation. *OsAAT* genes on duplicated chromosomal segments were explored by searching the segmental genome duplication of rice at RGAP segmental duplication database (http://rice.plantbiology.msu.edu/segmental_dup/500 kb/segdup_500 kb.shtml). Genes distributed nearby and separated by five or fewer genes were considered to be tandem duplicates.

### Phylogenetic Analysis and Sequence Alignment

The multiple sequence alignment was performed by using ClustalX version 1.83, and the unrooted phylogenetic tree based on the protein sequences of OsAAT members was constructed by neighbor-joining method. Bootstrap analysis was performed by using 1000 replicates. MEGA software version 4 [67] was used to display the phylogenetic tree. With the same method, the combined tree with OsAAT and AtAAT proteins was generated. The phylogeny of 85 OsAATs was also constructed by using the Bayesian estimation of MrBayes program [68]. The command “mcmc ngen = 1200000 samplefreq = 1200” were executed. When the calculation was finished, “the average standard deviation of split frequencies” was 0.0087. The program was stopped by typing “sump burnin = 250” and “sumt burnin = 250” and the phylogenetic tree was visualized through Treeview program.

The MEME motif search tool (http://meme.sdsc.edu/meme/intro.html) was used to identify motifs shared among related proteins within *OsAAT* gene family with default settings except that the maximum number of motifs was defined as 20 and the maximum width was set to 300. The sequence conservation of OsAAT subfamily members in amino acid was analyzed by DNAMAN software [52] and modified manually, and the conserved motifs and TM domains were annotated according to MEME analysis and TMHMM prediction, respectively.

### Expression Data Analysis of *OsAATs*

The EST, microarrays and MPSS expression data of *OsAAT* genes were extracted from UniGene database at NCBI (http://www.ncbi.nlm.nih.gov/unigene/), the Rice Functional Genomic Express Database (http://signal.salk.edu/cgi-bin/RiceGE) and the MPSS project (http://mpss.udel.edu), respectively. On the other hand, the expression data of *AtAATs* comparable to those used for rice were also downloaded (Table S3, S4 and S5). EST and MPSS data were used to detect expression pattern of *OsAATs* in different organs. The data of microarrays were used to analyze expression profiles of *OsAATs* in organs during different development stages (GSE6893) and under abiotic stresses (GSE6901). The absolute signal values were respectively divided by the average of all absolute values. The Cluster and Treeview software [68] were used to generate hierarchical cluster displays using the logarithmic values of the ratios in previous step. In expression comparative analysis of *OsAATs* and *AtAATs*, the genes that were up- or down-regulated at least two-fold with P<0.05 were considered to be differentially expressed.

### Quantitative Real-time PCR Analysis

To confirm the expression of representative *OsAAT* genes in rice organs, quantitative real-time PCR analysis was performed by SYBR-green fluorescence using gene-specific primers (Table S8). Primers were checked using dissociation curve analysis after the PCR reaction for their specificity. At least two or three independent biological replicates were made for real-time PCR analysis in different organs and under stress treatments, respectively. Three technical replicates were taken in each biological replicate. The first-strand cDNA was synthesized from DNaseI-treated total RNA using reverse transcriptase (ReverTra Ace, TOYOBO); 10-fold diluted cDNA samples were used for qRT-PCR. *TransStart* Eco Green qPCR SuperMix (TransGen, CHINA) was used to determine the expression levels for the genes in a Rotor-Gene Q real-time PCR machine (Qiagen). The following program was used: 9?°C for 30 s; 40 cycles of 95°C for 10 s, 55°C for 15 s, 72°C for 10 s. Rice *UBQ5* was used as an internal control gene [69]. The relative expression levels were analyzed by the standard curve method, three times diluted series of a mixed cDNA pools were selected to build a stand curve for each gene [68]. The given values of these diluted series are 1, 3, 9 and 27 (from low to high).

## Supporting Information

Figure S1 Structures of *OsAAT* genes. Gene structure analysis for 85 *OsAATs* is performed by using GSDS (http://gsds.cbi.pku.edu.cn/). The untranslated-regions (UTR), exons and introns are represented by gray boxes, black boxes and lines, respectively.
(TIF)

Figure S2 Prediction of the transmembrane regions of 85 OsAATs. The transmembrane regions of 85 OsAATs were predicted by using the TMHMM Server v2.0 (http://www.cbs.dtu.dk/services/TMHMM/) and displayed according to the order in Table 1.
(TIF)

Figure S3 **Chromosomal localization and duplication of** ***AtAAT*** **genes.** Chromosome numbers are indicated at the top of each bar. The scale on the left is in megabases (Mb). The cleavages on the chromosomes (vertical bars) indicate the position of centromeres.
(TIF)

Figure S4 **Bayesian phylogenetic analysis of OsAATs using MrBayes program.** Numbers at the nodes are posterior probability for MrBayes reconstructions. The numbers in AAP subfamily are marked by the letters.
(TIF)

Figure S5 **Phylogenetic analysis of OsAATs and AtAATs.** The phylogenetic tree of all AATs from *Arabidopsis* and rice after multiple sequence alignment using the full-length protein sequences is constructed by neighbor-joining method. The branches of different subfamilies are marked by different colors.
(TIF)

Table S1 **The general information and sequence characterization of 63** ***AtAAT*** **genes.**
(DOC)

Table S2 **The MEME motif sequences and lengths in OsAAT proteins.**
(DOC)

Table S3 **The EST expression profiles of** ***OsAAT*** **genes.**
(DOC)

Table S4 **The microarray analysis of** ***OsAAT*** **genes in various organs under abiotic stresses.**
(DOC)

Table S5 **The MPSS analysis of** ***OsAAT*** **genes.**
(DOC)

Table S6 **Data for expression comparison of** ***OsAAT*** **and** ***AtAAT*** **genes.**
(DOC)

Table S7 **Correlation coefficients between expression patterns from homologous genes.**
(DOC)

Table S8 **Primers used in qRT-PCR of** ***OsAAT*** **genes.**
(DOC)
