# Supplementary figures and images for: Genome-Wide Survey and Expression Analysis of Amino Acid Transporter Gene Family in Rice (Oryza sativa L.)
Source: PLoS One. 2012 Nov 15;7(11):e49210. doi: 10.1371/journal.pone.0049210 (PMC3499563; doi:10.1371/journal.pone.0049210)

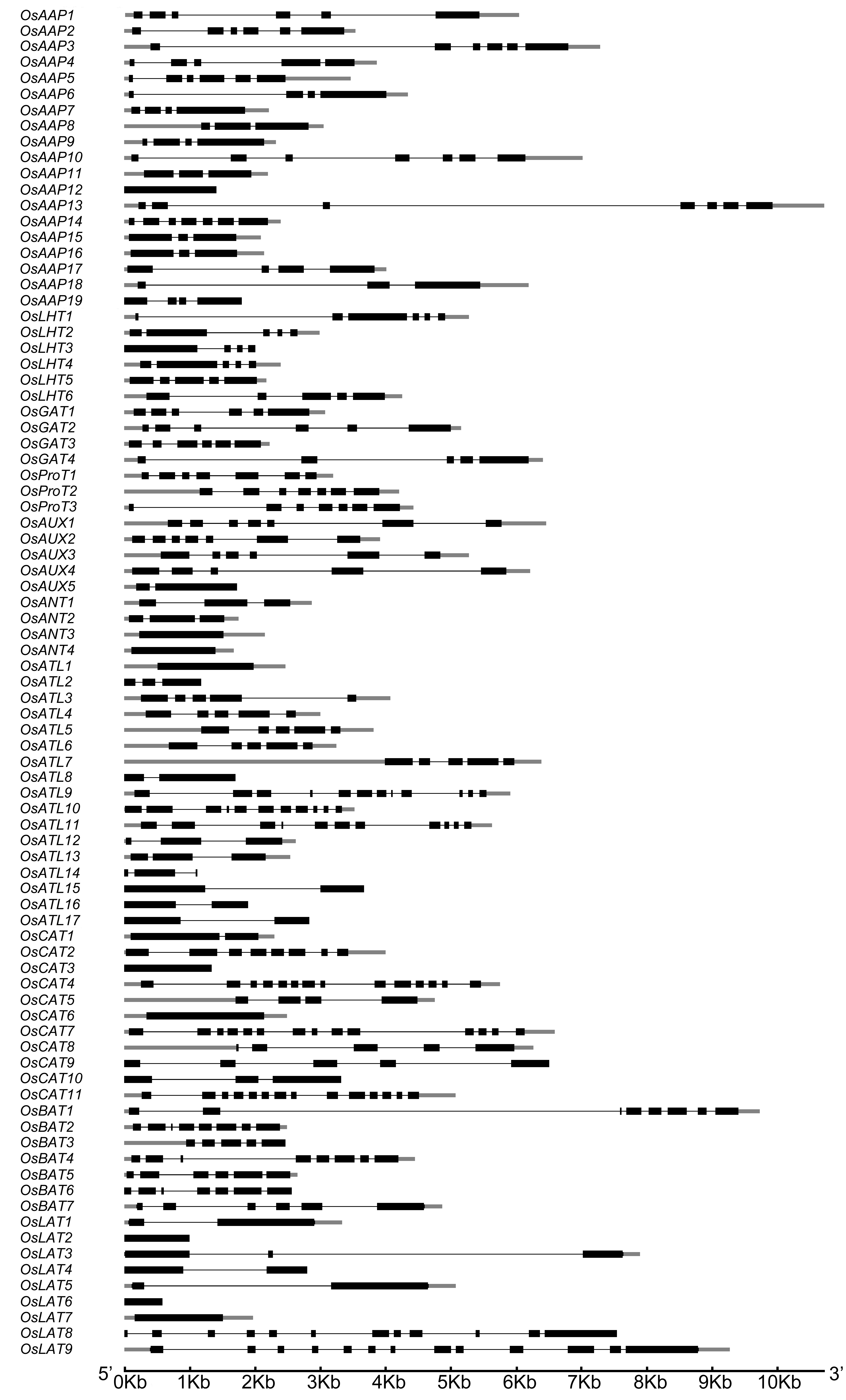

Supplement: Figure S1 — Structures of OsAAT genes. Gene structure analysis for 85 OsAATs is performed by using GSDS (http://gsds.cbi.pku.edu.cn/). The untranslated-regions (UTR), exons and introns are represented by gray boxes, black boxes and lines, respectively. (TIF) [file pone.0049210.s001.tif]

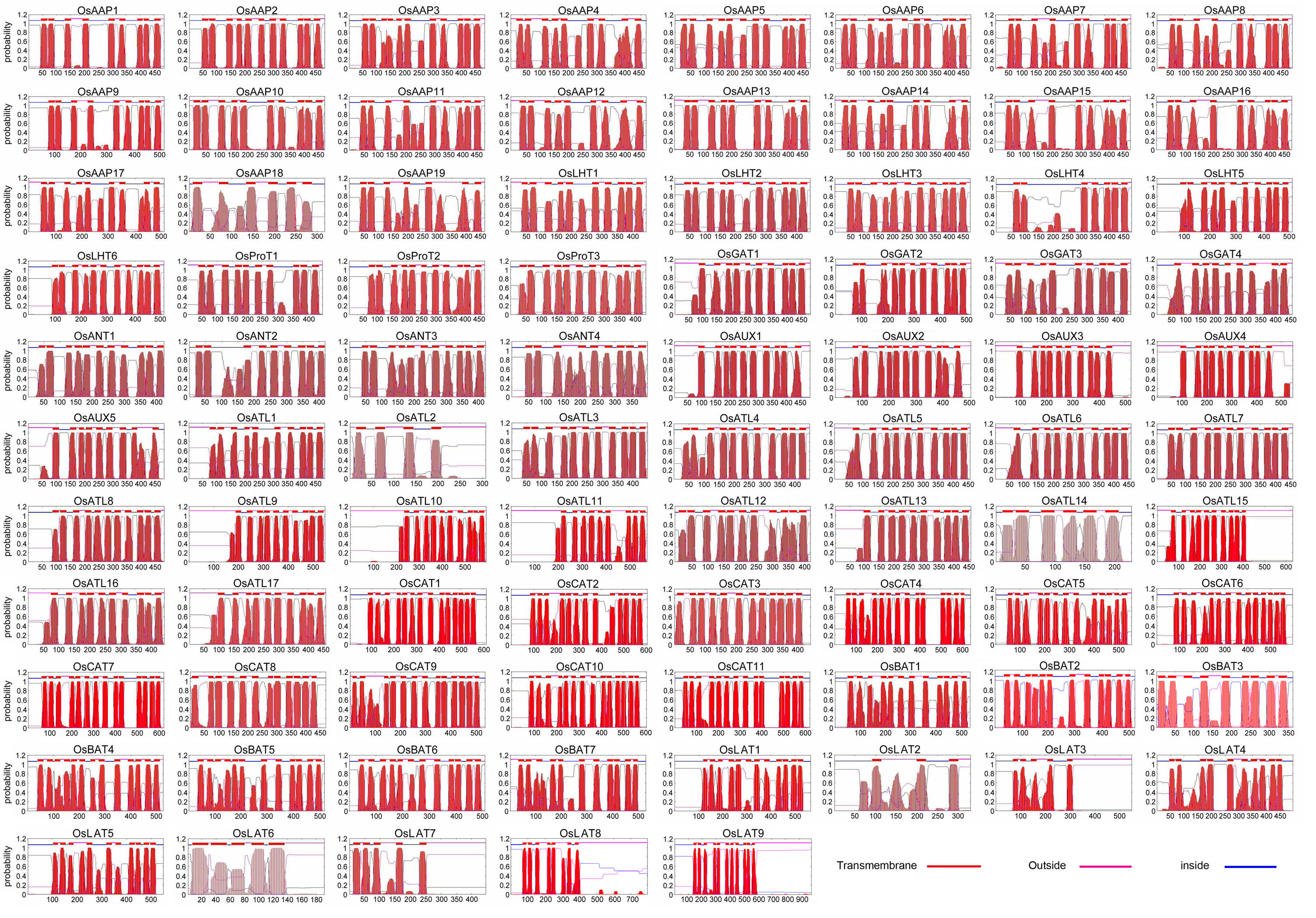

Supplement: Figure S2 — Prediction of the transmembrane regions of 85 OsAATs. The transmembrane regions of 85 OsAATs were predicted by using the TMHMM Server v2.0 (http://www.cbs.dtu.dk/services/TMHMM/) and displayed according to the order in Table 1. (TIF) [file pone.0049210.s002.tif]

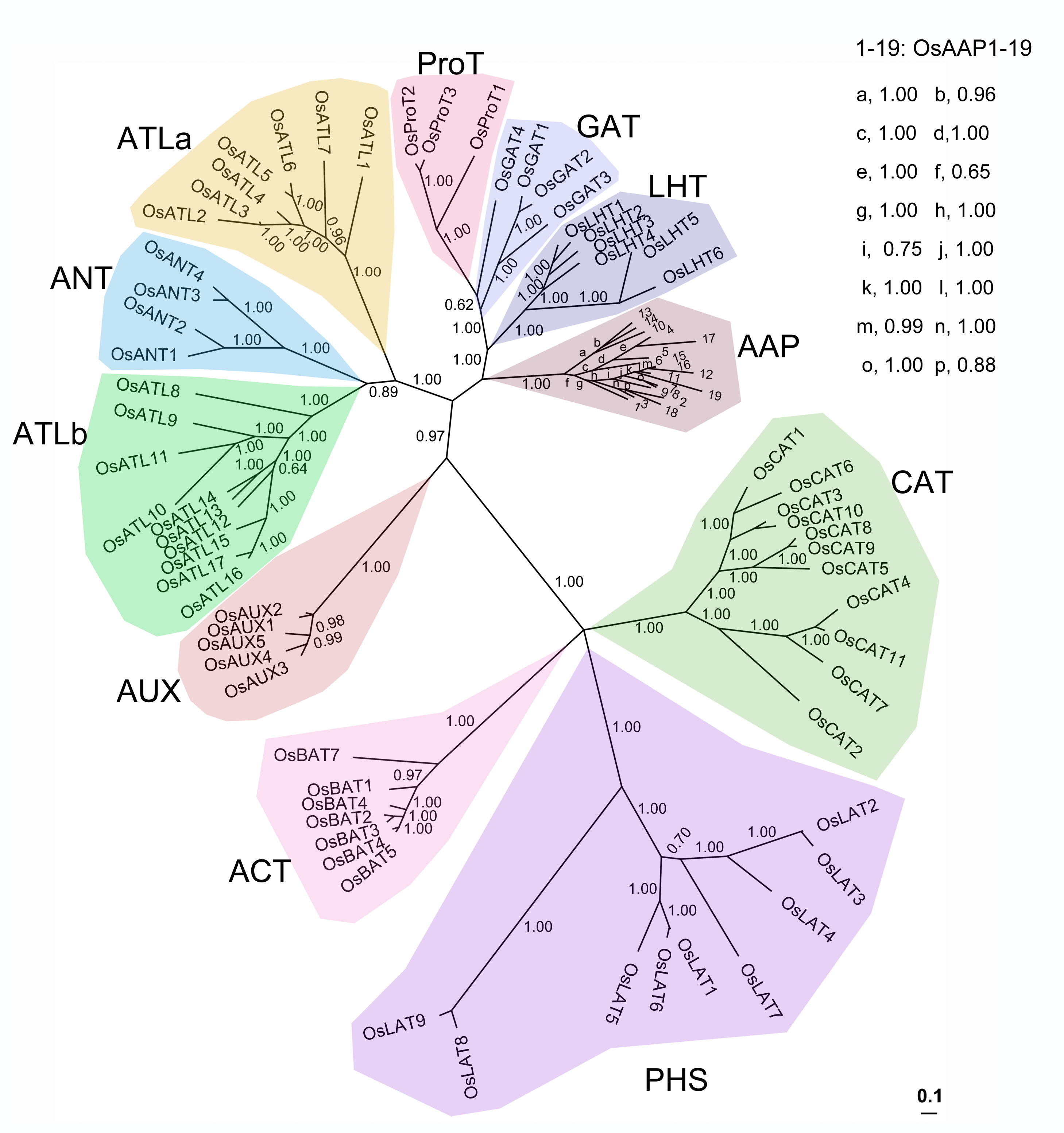

Supplement: Figure S4 — Bayesian phylogenetic analysis of OsAATs using MrBayes program. Numbers at the nodes are posterior probability for MrBayes reconstructions. The numbers in AAP subfamily are marked by the letters. (TIF) [file pone.0049210.s004.tif]
