# Supplementary material for: Genome-Wide Survey and Expression Analysis of Amino Acid Transporter Gene Family in Rice (Oryza sativa L.)
Source: PLoS One. 2012 Nov 15;7(11):e49210. doi: 10.1371/journal.pone.0049210 (PMC3499563; doi:10.1371/journal.pone.0049210)
Supplement: Table S1 — The general information and sequence characterization of 63 AtAAT genes. (DOC) [file pone.0049210.s006.doc]

**Table S1. The general information and sequence characterization of 63 *AtAAT* genes.**

| **S.N.** | **Gene^a^** | **Locus^b^** | **Gene Structure** | | **ORF(bp)^e^** | **Protein^f^** | | | **TM region^g^** | **expression^h^** |
| --- | --- | --- | --- | --- | --- | --- | --- | --- | --- | --- |
|  |  |  | **Length(bp)^c^** | **Intron^d^** |  | **Size (aa)** | **MW(d)** | **pI** |  |  |
|  | AAP group |  |  |  |  |  |  |  |  |  |
| 1 | *AtAAP1* | AT1G58360 | 3973 | 5 | 1458 | 485 | 52894.9 | 8.9626 | 9 | A C D |
| 2 | *AtAAP2* | AT5G09220 | 2803 | 6 | 1482 | 493 | 54146.9 | 9.1567 | 9 | A B C D |
| 3 | *AtAAP3* | AT1G77380 | 2503 | 6 | 1431 | 476 | 52036.6 | 9.0651 | 9 | A B C D |
| 4 | *AtAAP4* | AT5G63850 | 2466 | 6 | 1401 | 466 | 51428.1 | 9.2909 | 9 | A B C D |
| 5 | *AtAAP5* | AT1G44100 | 3281 | 4 | 1443 | 480 | 52537.9 | 8.3773 | 10 | A B C D |
| 6 | *AtAAP6* | AT5G49630 | 4029 | 5 | 1446 | 481 | 53020.3 | 8.6231 | 10 | A C D |
| 7 | *AtAAP7* | AT5G23810 | 2439 | 6 | 1404 | 467 | 51722.3 | 8.6986 | 10 | A C D |
| 8 | *AtAAP8* | AT1G10010 | 2751 | 5 | 1428 | 475 | 51814.9 | 9.1638 | 9 | A B C |
|  | LHT group |  |  |  |  |  |  |  |  |  |
| 9 | *AtLHT1* | AT5G40780 | 3631 | 7 | 1341 | 446 | 49813.3 | 8.9584 | 11 | A B C D |
| 10 | *AtLHT2* | AT1G24400 | 2422 | 6 | 1326 | 441 | 49151.9 | 8.8717 | 9 | A C |
| 11 | *AtLHT3* | AT1G61270 | 2476 | 7 | 1356 | 451 | 50325.1 | 9.4225 | 11 | A C |
| 12 | *AtLHT4* | AT1G47670 | 2872 | 4 | 1560 | 519 | 57118.5 | 9.5489 | 10 | A B C D |
| 13 | *AtLHT5* | AT1G67640 | 1781 | 5 | 1326 | 441 | 49420.1 | 9.1853 | 9 | A C |
| 14 | *AtLHT6* | AT3G01760 | 1972 | 7 | 1368 | 455 | 50735.5 | 8.752 | 11 | A C |
| 15 | *AtLHT7* | AT4G35180 | 2127 | 4 | 1437 | 478 | 52293.8 | 9.1328 | 11 | A B C D |
| 16 | *AtLHT8* | AT1G71680 | 2602 | 7 | 1347 | 448 | 49794.0 | 8.9645 | 9 | A C D |
| 17 | *AtLHT9* | AT1G25530 | 2885 | 6 | 1323 | 440 | 48815.8 | 8.9671 | 8 | A B C D |
| 18 | *AtLHT10* | AT1G48640 | ND | 8 | 1362 | 453 | 50449.1 | 7.8567 | 11 | A C D |
|  | GAT group |  |  |  |  |  |  |  |  |  |
| 19 | *AtGAT1* | AT1G08230 | 3091 | 5 | 1356 | 451 | 49694.3 | 8.5822 | 10 | A B C D |
| 20 | *AtGATL1* | AT5G41800 | 2219 | 6 | 1359 | 452 | 49855.6 | 8.9478 | 10 | A B C D |
|  | ProT group |  |  |  |  |  |  |  |  |  |
| 21 | *AtProT1* | AT2G39890 | 2653 | 6 | 1329 | 442 | 48455.5 | 9.7583 | 11 | A C D |
| 22 | *AtProT2* | AT3G55740 | 2767 | 6 | 1320 | 439 | 48085.2 | 9.4454 | 11 | A B C D |
| 23 | *AtProT3* | AT2G36590 | 2332 | 6 | 1311 | 436 | 47650.9 | 9.7987 | 11 | A B C D |
|  | AUX group |  |  |  |  |  |  |  |  |  |
| 24 | *AtAUX1* | AT2G38120 | 4188 | 8 | 1458 | 485 | 54059.4 | 8.4416 | 10 | A B C D |
| 25 | *AtLAX1* | AT5G01240 | 3942 | 8 | 1467 | 488 | 54600.2 | 8.8285 | 10 | A C D |
| 26 | *AtLAX2* | AT2G21050 | 2547 | 5 | 1452 | 483 | 54702.5 | 8.7508 | 10 | A B C D |
| 27 | *AtLAX3* | AT1G77690 | 2400 | 6 | 1413 | 470 | 53305.0 | 9.153 | 10 | A B C D |
|  | ANT group |  |  |  |  |  |  |  |  |  |
| 28 | *AtANT1* | AT3G11900 | 1927 | 2 | 1299 | 432 | 46469.9 | 5.1587 | 10 | A B C D |
| 29 | *AtANT2* | AT5G65990 | 1632 | 1 | 1284 | 427 | 46248.2 | 7.4964 | 11 | A B C D |
| 30 | *AtANT3* | AT4G38250 | 1667 | 0 | 1311 | 436 | 47210.4 | 6.6581 | 10 | A B C D |
| 31 | *AtANT4* | AT2G42005 | 1391 | 0 | 1242 | 413 | 44961.3 | 7.9869 | 9 | A D |
|  | ATLa group |  |  |  |  |  |  |  |  |  |
| 32 | *AtT1* | AT2G40420 | 1992 | 4 | 1323 | 440 | 47669.6 | 6.7845 | 11 | A B C D |
| 33 | *AtT2* | AT3G56200 | 1999 | 4 | 1308 | 435 | 47104.1 | 8.5898 | 11 | A B C D |
| 34 | *AtT3* | AT3G30390 | 2785 | 5 | 1383 | 460 | 49516.5 | 6.5027 | 11 | A B C D |
| 35 | *AtT4* | AT5G38820 | 1702 | 4 | 1371 | 456 | 49677.7 | 5.8962 | 11 | A C D |
| 36 | *AtT5* | AT1G80510 | 1945 | 0 | 1470 | 489 | 52957.5 | 6.3133 | 11 | A C D |
|  | ATLb group |  |  |  |  |  |  |  |  |  |
| 37 | *AtVAAT1* | AT3G28960 | 1523 | 2 | 1218 | 405 | 44643.5 | 8.9409 | 9 | A C D |
| 38 | *AtVAAT2* | AT5G15240 | 2674 | 2 | 1272 | 423 | 46472.3 | 7.8944 | 10 | A C D |
| 39 | *AtVAAT3* | AT2G41190 | 3129 | 10 | 1611 | 536 | 58862.3 | 4.5168 | 11 | A B C D |
| 40 | *AtVAAT4* | AT2G39130 | 4311 | 11 | 1653 | 550 | 60068.8 | 5.5766 | 9 | A B C D |
| 41 | *AtVAAT5* | AT3G54830 | 3130 | 11 | 1641 | 546 | 59735.6 | 5.4795 | 6 | A D |
| 42 | *AtVAAT6* | AT3G09330 | 2449 | 9 | 1575 | 524 | 57067.0 | 4.6133 | 10 | A C |
| 43 | *AtVAAT7* | AT3G09340 | 2477 | 9 | 1587 | 528 | 57451.4 | 4.527 | 10 | A C D |
| 44 | *AtVAAT8* | AT5G02170 | 2863 | 10 | 1581 | 526 | 57537.5 | 6.243 | 11 | A B C D |
| 45 | *AtVAAT9* | AT5G02180 | 2865 | 10 | 1653 | 550 | 59666.6 | 4.7823 | 9 | A B C D |
| 46 | *AtVAAT10* | AT5G16740 | 1813 | 2 | 1281 | 426 | 45807.7 | 8.9863 | 11 | A D |
|  | CAT group |  |  |  |  |  |  |  |  |  |
| 47 | *AtCAT1* | AT4G21120 | 3929 | 2 | 1785 | 594 | 64846.3 | 7.901 | 14 | A B C D |
| 48 | *AtCAT2* | AT1G58030 | 4420 | 13 | 1908 | 635 | 67114.6 | 5.8495 | 14 | A B C D |
| 49 | *AtCAT3* | AT5G36940 | 4219 | 14 | 1830 | 609 | 65284.0 | 7.8745 | 14 | A C D |
| 50 | *AtCAT4* | AT3G03720 | 5363 | 14 | 2406 | 801 | 86373.5 | 6.3673 | 15 | A B C D |
| 51 | *AtCAT5* | AT2G34960 | 1925 | 0 | 1710 | 569 | 62634.8 | 9.0476 | 14 | A C D |
| 52 | *AtCAT6* | AT5G04770 | 3352 | 3 | 1752 | 583 | 62808.0 | 8.7193 | 15 | A B C D |
| 53 | *AtCAT7* | AT3G10600 | NA | 3 | 1755 | 584 | 63701.5 | 6.7675 | 15 | A C D |
| 54 | *AtCAT8* | AT1G17120 | NA | 0 | 1773 | 590 | 64861.4 | 8.5624 | 13 | A B C D |
| 55 | *AtCAT9* | AT1G05940 | 2823 | 7 | 1710 | 569 | 60175.6 | 7.6647 | 14 | A B C D |
|  | ACT group |  |  |  |  |  |  |  |  |  |
| 56 | *AtBAT1* | AT2G01170 | 2356 | 7 | 1551 | 516 | 55331.1 | 8.1607 | 13 | A B C D |
|  | PHS group |  |  |  |  |  |  |  |  |  |
| 57 | *AtLAT1* | AT5G05630 | 1871 | 0 | 1473 | 490 | 53693.3 | 9.5351 | 12 | A B C D |
| 58 | *AtLAT2* | AT1G31820 | 1829 | 1 | 1449 | 482 | 52598.5 | 4.6396 | 9 | A B C D |
| 59 | *AtLAT3* | AT1G31830 | 2213 | 0 | 1488 | 495 | 54827.0 | 6.1575 | 10 | A B C D |
| 60 | *AtLAT4* | AT3G19553 | 2178 | 2 | 1440 | 479 | 52842.5 | 5.0961 | 12 | A C D |
| 61 | *AtLAT5* | AT3G13620 | 1880 | 1 | 1437 | 478 | 52734.6 | 7.8836 | 10 | A B C D |
|  | TTP group |  |  |  |  |  |  |  |  |  |
| 62 | *AtTTP1* | AT2G33260 | 1413 | 1 | 1311 | 436 | 46407.0 | 9.4725 | 11 | A C D |
| 63 | *AtTTP2* | AT5G19500 | 2994 | 13 | 1518 | 505 | 54356.9 | 4.949 | 10 | A B C D |

*^a^* Systematic designation given to Arabidopsis *AATs* in this study.

*^b^* Locus identity number of *AtAATs* assigned by TAIR.

*^c^* Gene full length of *AtAATs* obtained from TAIR.

*^d^* Number of intron in *AtAAT* genes from TAIR.

*^e^* Length of the open reading frame for *AtAATs*.

*^f^* Protein characterization of AtAATs obtained from TAIR.

*^g^* Number of transmembrane segments possessed by AtAATs, predicted by the TMHMM Server v2.0.

**^h^** Evidence for gene expression from (A) full-length cDNA, (B) ESTs, (C) microarray data, (D) massively parallel signature sequencing (MPSS).

S.N., serial number; ORF, open reading frame; bp, base pair; aa, amino acids; MW, molecular weight; pI, isoelectric point; TM, transmembrane; NA, not available.
