# Supplementary material for: Genome-Wide Survey and Expression Analysis of Amino Acid Transporter Gene Family in Rice (Oryza sativa L.)
Source: PLoS One. 2012 Nov 15;7(11):e49210. doi: 10.1371/journal.pone.0049210 (PMC3499563; doi:10.1371/journal.pone.0049210)
Supplement: Table S2 — The MEME motif sequences and lengths in OsAAT proteins. (DOC) [file pone.0049210.s007.doc]

## Table S2. The MEME motif sequences and lengths in OsAAT proteins.

| **Motif** | **Width** | **Conserved amino acid sequences** |
| --- | --- | --- |
| 1 | 56 | RNYTYMDAVRANLGGKKVWFCGCCQYVNLWGTMIGYTITASISMRAIKRANCFHRN |
| 2 | 29 | ACFHIITAVIGSGVLSLPWAMKQLGWVSG |
| 3 | 48 | KPPPSENKTMKKATMYSIATTTVFYMLCGCMGYAAFGNDAPGNLLTGF |
| 4 | 41 | EPYWLIDVANMCIVVHLVGAYQVYCQPIFEFMERWAARRWP |
| 5 | 159 | THFNTENGMGIHDKAYILAVGLLMSQYSMIGYDTSAHMIEETKNADWSGPIGIITSVALSTMFGWIYLIALTSIMTDIPYLLNPENDAGGYAIAQALYTAFHRRYGSGAGGIACLGVVAVAIFLCGIACITSNSRMAYAFSRDGAMPLSRVWHRVNKHE |
| 6 | 42 | FGVVQIFFSQIPNFHQIWWLSIVAAVMSFTYSTIGWGLCIAQ |
| 7 | 60 | VNPFRLTWRTAFVCATTVVAMLLPFFNDVVGLLGAVSFWPLTVYFPVEMYIAQRKIRRWS |
| 8 | 134 | GSWTAYLISILYVEYRTRKEREKVDFRNHVIQWFEVLDGLLGRHWRNAGLAFNCTFLLFGSVIQLIACASNIYYINDRLDKRTWTYIFGACCATTVFIPSFHNYRIWSFLGLLMTTYTAWYLAIAALIHGQVDG |
| 9 | 224 | YTFGGHAVTVEIMHAMWRPQKFKYIYLMATLYVLTLTLPSASAMYWAFGDELLTHSNAFALLPRTPWRDAAVILMLIHQFITFGFACTPLYFVWEKLIGMHGCRSICKRAAARLPIVVPIWFLAIIFPFFGPINSAVGSLLVSFTVYIIPALAHMITFRSAHARENAVEKPPRFLGGWTGMFVINCFVVAWVLVVGFGFGGWASMTNFIRQIDTFGLFTKCYQC |
| 10 | 21 | ALGDIAFAYSYHNILIEIQDT |
| 11 | 28 | FWNYNYWDNVSTMAEEVKNPQRDIPIGL |
| 12 | 105 | AKHGMQWAMYVVATGAVKGMCTTLMVSMLGQPRYLCHIARDHMMPPWFAKVHPRTQTPVNATIFMGCCTACIALFTDLDVLANMVSIGTLFIFMMVANALLYRRY |
| 13 | 75 | LWGIPTALGLYFFCYEGHCVFPNIYSSMKNRKKFPKVLLICFVICTLNYACMAVCGYLMYGEDTQSQVTLNLPEN |
| 14 | 96 | VPLNVVWLSVAVAFVMALTSLGSQVAFQAMVSIATIGMYIAYALPIFFRVTTARKSFVPGPFHLGKYGVVVGWVAVVWVATVTVLFSLPVAYPVAK |
| 15 | 21 | FACVCYYTSTLLADCYRSDDP |
| 16 | 29 | WNCCEALCMAEICSMYPTNGGYYYWSDKC |
| 17 | 70 | SYALHLMLVFPIVFHALRLNMDGLLFPSARPLSSDNRRFAIITAELLTVIYLAANFIPNIWDAFQFTGAT |
| 18 | 27 | RTAYVASTTFVACMFPFFGDFMSFIGS |
| 19 | 29 | RWVALQALSACCLVVSIAAAVGSIQDVID |
| 20 | 57 | MASKYVVLAIYAAILILHGIINSLPIQWLSWFGQLGAFWNAAGVFVLTILIPAVAKE |
