# Supplementary material for: Genome-Wide Survey and Expression Analysis of Amino Acid Transporter Gene Family in Rice (Oryza sativa L.)
Source: PLoS One. 2012 Nov 15;7(11):e49210. doi: 10.1371/journal.pone.0049210 (PMC3499563; doi:10.1371/journal.pone.0049210)
Supplement: Table S3 — The EST expression profiles of OsAAT genes. (DOC) [file pone.0049210.s008.doc]

**Table S3. The ESTs expression profiles of *OsAAT* genes.**

| **Gene** | **Locus** | **C** | **F** | **L** | **P** | **R** | **S** | **St** | **SAM** | **WP** |
| --- | --- | --- | --- | --- | --- | --- | --- | --- | --- | --- |
| *OsAAP1* | LOC_Os07g04180 | 169 | 7 | 64 | 440 | 117 | 92 | 55 | - | + |
| *OsAAP2* | LOC_Os06g12330 | - | - | - | 14 | - | - | - | - | + |
| *OsAAP3* | LOC_Os06g36180 | 72 | 36 | 34 | 43 | 161 | 30 | 31 | - | + |
| *OsAAP4* | LOC_Os12g09300 | 6 | 21 | 11 | 21 | 29 | - | 7 | - | + |
| *OsAAP5* | LOC_Os01g65660 | 30 | 14 | 29 | 50 | 14 | - | - | - | + |
| *OsAAP6* | LOC_Os01g65670 | 315 | 65 | 5 | 158 | 73 | 185 | 31 | - | + |
| *OsAAP7* | LOC_Os05g34980 | - | - | 40 | - | - | - | 31 | - | + |
| *OsAAP8* | LOC_Os01g66010 | - | - | - | 14 | 29 | - | 7 | - | + |
| *OsAAP9* | LOC_Os02g01210 | - | - | 5 | - | - | - | 7 | - | + |
| *OsAAP10* | LOC_Os02g49060 | - | 14 | - | 7 | - | 30 | - | - | + |
| *OsAAP11* | LOC_Os11g09020 | - | 139 | 40 | 93 | - | 61 | 126 | - | + |
| *OsAAP12* | LOC_Os12g09320 | - | - | - | - | - | - | - | - | - |
| *OsAAP13* | LOC_Os04g39489 | - | 58 | 34 | 79 | 146 | - | 7 | - | + |
| *OsAAP14* | LOC_Os04g56470 | **133** | - | - | 14 | 14 | - | 7 | - | + |
| *OsAAP15* | LOC_Os12g08130 | 66 | - | 75 | 325 | 102 | - | 79 | - | + |
| *OsAAP16* | LOC_Os12g08090 | 36 | 36 | 17 | 209 | - | 30 | 31 | - | + |
| *OsAAP17* | LOC_Os06g12350 | - | - | - | **50** | - | - | - | - | + |
| *OsAAP18* | LOC_Os06g36210 | 60 | 29 | - | - | - | - | - | - | + |
| *OsAAP19* | LOC_Os04g41350 | - | - | - | - | - | - | - | - | - |
| *OsLHT1* | LOC_Os08g03350 | 151 | 146 | 250 | 57 | 541 | - | 63 | - | + |
| *OsLHT2* | LOC_Os12g14100 | - | **175** | - | - | - | - | - | - | + |
| *OsLHT3* | LOC_Os05g14820 | - | - | - | - | - | - | - | - | - |
| *OsLHT4* | LOC_Os04g38860 | - | - | - | 14 | 73 | - | - | - | + |
| *OsLHT5* | LOC_Os04g47420 | - | - | 11 | 7 | - | - | 7 | - | + |
| *OsLHT6* | LOC_Os12g30040 | 36 | 21 | 46 | 7 | - | - | 15 | - | + |
| *OsGAT1* | LOC_Os05g50920 | - | - | - | 36 | 87 | - | - | - | + |
| *OsGAT2* | LOC_Os01g43320 | - | - | 23 | - | - | - | - | - | + |
| *OsGAT3* | LOC_Os10g27980 | 18 | - | 5 | - | - | - | - | - | + |
| *OsGAT4* | LOC_Os01g63854 | 24 | 51 | 116 | 50 | 87 | - | - | - | + |
| *OsProT1* | LOC_Os01g68050 | - | - | 52 | 14 | - | 61 | 31 | - | + |
| *OsProT2* | LOC_Os03g44230 | 91 | 14 | 17 | 14 | - | - | 71 | - | + |
| *OsProT3* | LOC_Os07g01090 | - | - | - | - | - | - | 15 | - | + |
| *OsAUX1* | LOC_Os01g63770 | 103 | 80 | 52 | 245 | 497 | 92 | 79 | - | + |
| *OsAUX2* | LOC_Os05g37470 | 6 | 14 | 23 | 93 | 43 | 61 | 55 | - | + |
| *OsAUX3* | LOC_Os03g14080 | **182** | - | - | 72 | - | - | - | - | + |
| *OsAUX4* | LOC_Os10g05690 | 72 | - | - | 21 | - | - | 7 | - | + |
| *OsAUX5* | LOC_Os11g06820 | - | - | - | 7 | - | - | - | - | + |
| *OsANT1* | LOC_Os07g12770 | 12 | 7 | - | 7 | - | - | 7 | - | + |
| *OsANT2* | LOC_Os03g60260 | 18 | - | 23 | - | 14 | - | 15 | - | + |
| *OsANT3* | LOC_Os02g44980 | 194 | 263 | 431 | 245 | 453 | 370 | 387 | - | + |
| *OsANT4* | LOC_Os04g47780 | - | 43 | 145 | 28 | 131 | - | 63 | - | + |
| *OsATL1* | LOC_Os06g43700 | 24 | 87 | 34 | 43 | - | - | - | - | + |
| *OsATL2* | LOC_Os09g26290 | - | - | - | - | - | - | - | - | - |
| *OsATL3* | LOC_Os02g49510 | - | 102 | - | 79 | - | - | - | - | + |
| *OsATL4* | LOC_Os06g16420 | 24 | 95 | 23 | 72 | - | 92 | 102 | - | + |
| *OsATL5* | LOC_Os06g42720 | 400 | 263 | 530 | 274 | 497 | 432 | 363 | - | + |
| *OsATL6* | LOC_Os02g09810 | 6 | 36 | 99 | 21 | 219 | 92 | - | - | + |
| *OsATL7* | LOC_Os01g61044 | 60 | - | 110 | 79 | - | - | 23 | - | + |
| *OsATL8* | LOC_Os11g19240 | - | - | - | - | - | - | - | - | - |
| *OsATL9* | LOC_Os02g54730 | 12 | - | - | 14 | 29 | - | 15 | - | + |
| *OsATL10* | LOC_Os12g38570 | - | 21 | - | - | - | - | - | - | + |
| *OsATL11* | LOC_Os02g01100 | 66 | 7 | 58 | 36 | 29 | 61 | 47 | - | + |
| *OsATL12* | LOC_Os06g12320 | - | 7 | 29 | 7 | - | - | - | - | + |
| *OsATL13* | LOC_Os04g38680 | 6 | 7 | 29 | 28 | - | - | 39 | - | + |
| *OsATL14* | LOC_Os04g38660 | - | - | - | - | - | - | - | - | - |
| *OsATL15* | LOC_Os01g41420 | - | - | **58** | 7 | - | - | - | - | + |
| *OsATL16* | LOC_Os01g41400 | - | - | - | - | - | - | - | - | - |
| *OsATL17* | LOC_Os01g40410 | - | - | - | - | - | - | - | - | - |
| *OsCAT1* | LOC_Os01g11160 | 6 | - | 5 | - | - | - | 7 | - | + |
| *OsCAT2* | LOC_Os02g43860 | 72 | - | 110 | 21 | - | - | 47 | - | + |
| *OsCAT3* | LOC_Os03g43970 | - | - | 11 | - | - | - | - | - | + |
| *OsCAT4* | LOC_Os03g45170 | 12 | - | 29 | 21 | 73 | 61 | 94 | - | + |
| *OsCAT5* | LOC_Os04g45950 | 24 | - | - | 7 | - | - | - | - | + |
| *OsCAT6* | LOC_Os06g34830 | 12 | 7 | - | 14 | 58 | - | - | - | + |
| *OsCAT7* | LOC_Os10g30090 | - | 65 | - | 14 | - | 30 | - | - | + |
| *OsCAT8* | LOC_Os11g05690 | 12 | - | 5 | - | - | - | - | - | + |
| *OsCAT9* | LOC_Os12g06060 | - | - | - | - | - | - | - | - | - |
| *OsCAT10* | LOC_Os12g41890 | - | - | - | - | - | - | - | - | - |
| *OsCAT11* | LOC_Os12g42850 | 84 | 29 | 559 | 43 | 146 | 123 | 173 | - | + |
| *OsBAT1* | LOC_Os01g42234 | 48 | 14 | 11 | 14 | - | 30 | 7 | - | + |
| *OsBAT2* | LOC_Os01g71700 | 12 | - | - | - | - | - | - | - | + |
| *OsBAT3* | LOC_Os01g71710 | - | - | - | - | - | - | - | - | - |
| *OsBAT4* | LOC_Os01g71720 | - | - | - | - | - | - | **86** | - | + |
| *OsBAT5* | LOC_Os01g71740 | 30 | 7 | 29 | - | 29 | 247 | - | - | + |
| *OsBAT6* | LOC_Os01g71760 | - | - | - | - | - | - | - | - | - |
| *OsBAT7* | LOC_Os04g35540 | 42 | 7 | 29 | 7 | 146 | - | 15 | - | + |
| *OsLAT1* | LOC_Os02g47210 | 78 | 21 | - | 43 | - | - | 31 | - | + |
| *OsLAT2* | LOC_Os03g25840 | - | - | - | - | - | - | - | - | - |
| *OsLAT3* | LOC_Os03g25869 | - | 14 | - | 101 | - | - | - | - | + |
| *OsLAT4* | LOC_Os03g25920 | - | - | 5 | 7 | - | - | - | - | + |
| *OsLAT5* | LOC_Os03g37984 | 139 | 564 | 87 | 115 | 29 | 123 | 102 | - | + |
| *OsLAT6* | LOC_Os08g41370 | - | - | - | - | - | - | - | - | - |
| *OsLAT7* | LOC_Os12g39080 | - | 7 | 69 | 7 | 14 | 30 | 7 | - | + |
| *OsLAT8* | LOC_Os01g19850 | 6 | 7 | - | 14 | - | 61 | - | - | + |
| *OsLAT9* | LOC_Os08g23440 | 12 | - | 56 | 58 | - | 61 | 24 | - | + |

C, callus; F, flower; L, leaf,; P, panicle; R, root; S, seed; St, stem; SAM, shoot apical meristem; WP, whole plant. Underlined and bold indicated specific expression; underlined indicated abundant expression. “+” and “-” represent “exist” and “not exist”, respectively.
