# Supplementary material for: Genome-Wide Survey and Expression Analysis of Amino Acid Transporter Gene Family in Rice (Oryza sativa L.)
Source: PLoS One. 2012 Nov 15;7(11):e49210. doi: 10.1371/journal.pone.0049210 (PMC3499563; doi:10.1371/journal.pone.0049210)
Supplement: Table S4 — The microarray analysis of OsAAT genes in various organs under abiotic stresses. (DOC) [file pone.0049210.s009.doc]

**Table S4. The microarray analysis of *OsAAT* genes in various organs and** under abiotic stresses.

| **Gene** | **Locus** | **Developmental stages** | | | | | | | | | | | | | | | **Abiotic stresses** | | | |
| --- | --- | --- | --- | --- | --- | --- | --- | --- | --- | --- | --- | --- | --- | --- | --- | --- | --- | --- | --- | --- |
|  |  | **YR** | **ML** | **YL** | **SAM** | **P1** | **P2** | **P3** | **P4** | **P5** | **P6** | **S1** | **S2** | **S3** | **S4** | **S5** | **CK** | **DS** | **SS** | **CS** |
| *OsAAP16* | Os12g08090 | 1.06 | 2.73 | 2.27 | -2.74 | 2.94 | 1.9 | 2.94 | 3.71 | 0.09 | 2.84 | 3.82 | 2.65 | 2.39 | 1.9 | -0.85 | 1.53 | 2.04 | 1.61 | 0.94 |
| *OsAAP15* | Os12g08130 | -0.97 | 1.63 | 1.94 | -1.82 | 2.4 | 1.42 | 2.37 | 3.19 | -0.24 | 2.71 | 3.31 | 1.8 | 1.29 | 0.11 | -2.88 | -0.3 | 0.89 | 0.75 | 0.8 |
| *OsATL9* | Os02g54730 | 0.62 | 2 | 2.18 | 0.46 | 0.51 | 0.37 | 0.69 | 1.11 | 1.12 | -0.15 | 1.18 | 0.87 | 0.91 | -0.33 | 0.69 | 2.17 | 0.75 | 1.68 | 1.83 |
| *OsLAT5* | Os03g37984 | 1.61 | 1.01 | 1.22 | 1.19 | 1.46 | 1.47 | 1.58 | 1.29 | 1.45 | 1.22 | 1.45 | 2.37 | 3.04 | 2.87 | 2.25 | 1.43 | 1.27 | 1.25 | 0.99 |
| *OsATL5* | Os06g42720 | 3.73 | 3.62 | 3.67 | 2.44 | 2.87 | 2.77 | 3.08 | 3.37 | 3.71 | 3.91 | 3.41 | 3.33 | 3.13 | 3.16 | 3.41 | 3.88 | 3.63 | 3.88 | 4.05 |
| *OsANT3* | Os02g44980 | 1.14 | 1.81 | 2.11 | 0.94 | 2.67 | 2.26 | 2.23 | 2.31 | 0.48 | 2.6 | 3.34 | 2.83 | 2.8 | 2.77 | 1.99 | 1.14 | 2.98 | 2.91 | 3.05 |
| *OsCAT11* | Os12g42850 | 3.11 | 3.18 | 3.99 | 0.28 | 1.2 | 0.91 | 1.47 | 1.8 | 1.45 | 2.89 | 2.52 | 2.34 | 2.57 | 2.98 | 3.18 | 2.9 | 3.51 | 3.71 | 3.17 |
| *OsATL6* | Os02g09810 | 0.73 | 2.63 | 3.03 | -0.64 | 1.83 | 0.77 | 2.09 | 1.88 | 0.56 | 2.49 | 2.46 | 2.39 | 2.34 | 2.56 | 2.19 | 0.61 | 3.8 | 3.68 | 2.11 |
| *OsLHT1* | Os08g03350 | 3.4 | 3.45 | 3.41 | -4.17 | 1.5 | -0.5 | 2.34 | 1.65 | 1.32 | 3.78 | 3.93 | 3.06 | 2.88 | 3.01 | 2.76 | 2.13 | 2.17 | 2.41 | 2.25 |
| *OsAUX1* | Os01g63770 | 2.44 | 0.8 | -0.4 | 0.88 | -0.03 | 0.43 | 0.97 | 1.4 | 3.01 | 1.12 | 1.1 | 0.19 | -0.06 | -0.38 | -0.91 | 1.51 | 0.69 | 0.74 | 0.61 |
| *OsATL4* | Os06g16420 | 0.93 | 2.1 | 1.57 | -2.82 | -1.24 | -1.6 | -0.72 | -0.14 | 0.84 | 1.58 | 1.47 | 0.87 | 1.2 | 1.32 | 0.84 | 1.42 | 1.61 | 1.73 | 1.53 |
| *OsAAP1* | Os07g04180 | 1.65 | 0.21 | -1.67 | 0.49 | -0.26 | 0.08 | 0.02 | 0.27 | 0.29 | 0.59 | 1.33 | 1.58 | 2.33 | 2.77 | 2.84 | 0.89 | 0.98 | 1.14 | 0.14 |
| *OsAUX2* | Os05g37470 | 0.94 | -0.61 | -1.69 | -1.85 | -1.22 | -1 | -0.66 | 0.08 | 0.23 | 0.66 | 0.02 | 0.4 | 0.1 | -0.42 | -1.07 | -0 | -1.54 | -1.34 | -0.46 |
| *OsAAP14* | Os04g56470 | -1.64 | -3.41 | -3.98 | -4.86 | -0.21 | 0.23 | 0.78 | 0.8 | -1.8 | 0.45 | -1.09 | -0.81 | -0.57 | -1.63 | -1.09 | -2.7 | -1.69 | -1.33 | -2.55 |
| *OsCAT2* | Os02g43860 | 0.36 | 0.29 | 0.61 | -1.9 | -0.61 | -0.8 | -0.34 | -0.38 | -0.45 | -0.22 | -0.02 | -0.54 | -0.41 | -0.81 | -0.83 | -0.5 | -0.69 | -0.57 | -0.54 |
| *OsAAP7* | Os05g34980 | 0.84 | 1.2 | 0.51 | -3.81 | -3.64 | -3.4 | -1.71 | -1.44 | -1.41 | 0.05 | -0.6 | -0.73 | -1.59 | -2.6 | -5.25 | 0.47 | 0.09 | -0.2 | 0.13 |
| *OsAAP4* | Os12g09300 | 1.51 | -1.04 | -1.26 | -2.5 | -3.31 | -2.6 | -2.8 | -2.22 | -1.21 | -0.97 | -2.02 | -1.46 | -1.99 | -2.68 | -4.74 | 0.11 | -2.38 | -1.33 | -0.82 |
| *OsCAT7* | Os10g30090 | -0.7 | -0.69 | -0.69 | -2.19 | -2.08 | -2.3 | -1.94 | -1.68 | -1.49 | -0.81 | -0.94 | -1.41 | -1.91 | -2.2 | -1.99 | -0.8 | -1.5 | -1.44 | -0.71 |
| *OsProT3* | Os07g01090 | 0.39 | -1.29 | -1.34 | -5.21 | -5.55 | -5 | -4.9 | -4.63 | -2.34 | -3.49 | -2.81 | -3.43 | -3.7 | -5.21 | -6.29 | 0.33 | -1.71 | -0.69 | -0.03 |
| *OsATL12* | Os06g12320 | 0.96 | -1.25 | -4.31 | -3.28 | -5.49 | -4.7 | -4.66 | -3.63 | 2.34 | 1.38 | -3.7 | -5.12 | -3.27 | -4.66 | -4.91 | -2.3 | -3.3 | -3.19 | -3.35 |
| *OsATL11* | Os02g01100 | 0.03 | -2.54 | -0 | -1.13 | -1.13 | -0.8 | -1.2 | -0.88 | -1.01 | -0.69 | -0.66 | -1.26 | -2.56 | -4.04 | -2.87 | -0.9 | -0.72 | 0.1 | -0.97 |
| *OsATL1* | Os06g43700 | -0.2 | -0.62 | -0.76 | -0.43 | -0.43 | -0.9 | -0.13 | 0 | -0.66 | 0.27 | -0.39 | -0.93 | -0.85 | -1.54 | -2.06 | -0.6 | -0.71 | -0.54 | -2.07 |
| *OsAUX5* | Os11g06820 | -0.58 | -3.5 | -3.23 | -3.37 | -3.28 | -2.8 | -1.06 | -0.22 | -0.43 | -2.6 | -3.03 | -3.96 | -4.61 | -4.36 | -3.75 | -1.3 | -1.03 | -1.38 | -2.47 |
| *OsAAP2* | Os06g12330 | -5.72 | -4.78 | -5.05 | -5.46 | -5.6 | -4.9 | -3.44 | -1.35 | -2.02 | -3.74 | -5.5 | -4.04 | -6.34 | -7.75 | -6.91 | -5.4 | -5.04 | -5.7 | -5.53 |
| *OsCAT4* | Os03g45170 | 0.35 | 0.49 | -0.04 | -0.28 | -0.28 | -1.3 | -0.36 | -0.51 | -1.41 | -0.57 | -2.85 | -1.84 | -1.66 | -2.4 | -1.98 | 1.45 | 1.36 | 1.14 | 1.43 |
| *OsLHT5* | Os04g47420 | -1.17 | -0.14 | -3.25 | -0.63 | -1.96 | -1.7 | -1.92 | -2.27 | -2.61 | -2.53 | -0.27 | -3.6 | -4.81 | -3.35 | -2.69 | -1.9 | -3.83 | -3.07 | -2.32 |
| *OsAAP8* | Os01g66010 | -0.35 | -0.63 | -2.13 | -2.49 | -1.99 | -2.2 | -2.14 | -1.56 | -2.78 | -2.74 | -1.73 | -3.95 | -3.64 | -3.98 | -3.84 | -0.3 | -1.96 | -1.61 | -1.32 |
| *OsCAT5* | Os04g45950 | -1.41 | -3.84 | -3.47 | -1.67 | -3.49 | -3.6 | -3.84 | -3.83 | -4.1 | -4.13 | -3.92 | -4.08 | -3.4 | -3.29 | -6.41 | -3.1 | -3.33 | -3.33 | -3.04 |
| *OsAUX3* | Os03g14080 | -2.95 | -3.77 | -3.68 | 0.22 | -1.96 | -1.8 | -2.74 | -3.4 | -3.56 | -4.46 | -4.38 | -4.43 | -3.75 | -3.76 | -3.72 | -3.6 | -3.95 | -3.72 | -3.74 |
| *OsBAT4* | Os01g71720 | -4.16 | 2.62 | 0.26 | -4.38 | -4.54 | -4.1 | -4.65 | -4.34 | -4.7 | -4.77 | -2.93 | -3.61 | -3.88 | -3.04 | -2.98 | -0.9 | -4.14 | -2.3 | -2.39 |
| *OsBAT1* | Os01g42234 | -0.01 | -1.02 | -2.09 | -2.75 | -1.96 | -2.7 | -2.28 | -1.4 | -2.78 | -1.4 | -0.49 | -2.18 | -0.9 | -0.91 | -1.76 | -1.1 | -0.67 | -1.2 | -1.29 |
| *OsLAT1* | Os02g47210 | -1.11 | -2.1 | -2.94 | -4.46 | -4.78 | -4.4 | -3.69 | -2.78 | -2.68 | -3.2 | -1.56 | -1.84 | -0.59 | -1.01 | -1.84 | -2.6 | -2.54 | -2.39 | -3.05 |
| *OsGAT3* | Os10g27980 | 0.7 | -4.2 | -2.24 | -4.76 | -4.52 | -4.5 | -4.62 | -5.6 | -4.9 | -3.83 | -2.78 | -2.73 | -2.56 | -2.33 | -1.88 | -1.7 | -3.42 | -3.83 | -2.13 |
| *OsLHT4* | Os04g38860 | 0.33 | -3.46 | -3.3 | -3.3 | -3.17 | -3.5 | -3.26 | -3.07 | -3.33 | -3.46 | -3.65 | -3.27 | -3.27 | -2.32 | -2.11 | -1.4 | -2 | -2.01 | -1.62 |
| *OsAAP10* | Os02g49060 | -1.21 | -3.07 | -3.66 | -4.49 | -4.56 | -4.3 | -3.99 | -4.21 | -2.54 | -3.2 | -3.11 | -3.11 | -2.45 | -1.66 | -3.84 | -2.5 | -3.26 | -3.31 | -2.75 |
| *OsLAT4* | Os03g25920 | -0.47 | -1.63 | -3.02 | -2.62 | -2.17 | -3.2 | -1.88 | -1.52 | -1.49 | -0.38 | -0.19 | -1.73 | -1.93 | -1.52 | -2.23 | -0.8 | -1.14 | -0.47 | -2.11 |
| *OsGAT4* | Os01g63854 | -0.05 | -1.63 | -3.38 | -4.22 | -2.3 | -3.2 | -2.02 | -1.34 | -3.47 | -1.29 | -1.05 | -2.35 | -2.42 | -2.49 | -4.04 | -1.6 | -1.45 | -1.4 | -1.81 |
| *OsAAP5* | Os01g65660 | -0.65 | -1.28 | -2.14 | -3.34 | -3.24 | -2.7 | -1.63 | -1.55 | -1.77 | -0.97 | -2.15 | -2.5 | -2.44 | -1.97 | -2.2 | -1.7 | -0.94 | -0.36 | -1.94 |
| *OsCAT9* | Os12g06060 | -1.51 | -2.24 | -3.02 | -1.92 | -2.72 | -3.2 | -2.45 | -1.64 | -1.18 | -1.74 | -1.99 | -2.3 | -1.13 | -0.88 | -0.59 | -1.2 | -1.65 | -1.99 | -1.65 |
| *OsGAT1* | Os05g50920 | -0.98 | -5.06 | -4.29 | -5.12 | -5.57 | -5.1 | -4.62 | -2.48 | -2.93 | -3 | -4.71 | -3.22 | -1.54 | 0.09 | -0.78 | -3.7 | -3.66 | -3.44 | -2.47 |
| *OsCAT6* | Os06g34830 | -3.06 | -3.12 | -3.51 | -3.84 | -3.59 | -3.5 | -2.74 | -1.11 | -0.8 | -0.54 | -1.67 | -2.01 | -0.6 | -0.82 | -1.68 | -3.1 | -1.67 | -2.25 | -3.51 |
| *OsLHT3* | Os05g14820 | -5.36 | -4.42 | -4.7 | -5.19 | -4.85 | -4.8 | -4.62 | -4.59 | -3.1 | -1.73 | -4.03 | -4.56 | -4.29 | -1.16 | -1.23 | -6 | -4.16 | -4.26 | -5.57 |
| *OsAAP18* | Os06g36210 | -2.07 | -4.21 | -4.53 | -4.93 | -3.8 | -3 | -3.4 | -2.97 | -2.93 | -0.73 | -3.21 | -4.44 | -3.77 | -2.6 | -1.19 | -4 | -3.35 | -3.3 | -3.59 |
| *OsLHT2* | Os12g14100 | -4.17 | -4.19 | -4 | -4.7 | -4.53 | -4.1 | -4.62 | -5.08 | -3.31 | 0.92 | -3.95 | -4.75 | -4.5 | -4.69 | -2.91 | -4.2 | -4.59 | -4.15 | -4.58 |
| *OsAAP17* | Os06g12350 | -5.41 | -4.97 | -4.69 | -5.33 | -5.44 | -5.2 | -5.48 | -4.89 | -0.52 | 0.23 | -4.7 | -5.87 | -6.14 | -4.89 | -4.74 | -5.6 | -5.23 | -5.52 | -5.39 |
| *OsATL10* | Os12g38570 | -4.18 | -4.09 | -4.07 | -3.99 | -4.15 | -4.1 | -3.47 | -4.15 | -3.37 | -0.99 | -4.2 | -3.89 | -4.32 | -3.17 | -4.83 | -4.2 | -4.43 | -4.1 | -4.19 |
| *OsBAT5* | Os01g71740 | -4.7 | -2.94 | -3 | -4.29 | -3.93 | -4 | -4.69 | -4.53 | -4.71 | -4.63 | -3.66 | -4.37 | -4.36 | -3.69 | -4.64 | -4.3 | -4.03 | -4.19 | -4.57 |
| *OsATL15* | Os01g41420 | -3.48 | -1.45 | -2.72 | -3.81 | -3.16 | -3.8 | -3.41 | -3.22 | -4.29 | -3.52 | -3.12 | -3.94 | -3.43 | -2.07 | -2.53 | -3.5 | -2.87 | -3.18 | -2.85 |
| *OsAAP9* | Os02g01210 | -3.62 | -0.99 | -2.37 | -4.17 | -4.05 | -4.1 | -4.15 | -3.7 | -3.79 | -3.91 | -3.26 | -3.12 | -3.13 | -2.97 | -3.33 | -1.6 | -3.39 | -2.42 | -2.04 |
| *OsBAT6* | Os01g71760 | -2.75 | -2.33 | -3.48 | -4.79 | -3.62 | -4.1 | -3.92 | -4.27 | -3.18 | -2.09 | -3.15 | -3.72 | -4.2 | -3.79 | -4.43 | -3.8 | -2.92 | -3.4 | -3.25 |
| *OsProT1* | Os01g68050 | -3.37 | -4.24 | -4.4 | -5.22 | -5.39 | -4.7 | -5.18 | -5.08 | -3.3 | -4.9 | -4.06 | -5.18 | -4.43 | -5.65 | -4.62 | -2.5 | -3.96 | -3.4 | -2.5 |
| *OsATL7* | Os01g61044 | -5.67 | -4.78 | -4.96 | -5.43 | -5.15 | -5.2 | -5.76 | -5.85 | -5.7 | -5.57 | -6.02 | -6.14 | -6.15 | -6.79 | -8.93 | -5.6 | -5.38 | -5.14 | -5.63 |
| *OsATL2* | Os09g26290 | -5.51 | -5.08 | -4.36 | -5.23 | -5.33 | -4.7 | -5.37 | -5.87 | -5.52 | -5.53 | -5.66 | -5.43 | -5.95 | -5.29 | -8.64 | -5.6 | -5.09 | -5.06 | -6.04 |
| *OsBAT2* | Os01g71700 | -4.85 | -4.13 | -3.58 | -4.53 | -4.34 | -5 | -4.53 | -5.5 | -5.43 | -5.1 | -4.13 | -4.1 | -4.84 | -5.76 | -6.16 | -4.2 | -4.94 | -4.25 | -5.26 |
| *OsLAT9* | Os08g23440 | -3.88 | -4.36 | -4.13 | -3.07 | -3.47 | -4.2 | -3.88 | -3.81 | -3.88 | -4.65 | -3.79 | -4.72 | -4.9 | -4.45 | -3.62 | -3.9 | -3.78 | -3.79 | -4.27 |
| *OsLAT8* | Os01g19850 | -3.85 | -3.63 | -4.15 | -2.91 | -3.41 | -3.4 | -3.55 | -3.05 | -2.07 | -2.87 | -3.25 | -3.4 | -3.51 | -3.28 | -3.19 | -4.5 | -4.6 | -4.37 | -4.37 |
| *OsAAP19* | Os04g41350 | -3.54 | -4.04 | -4.56 | -5.07 | -4.51 | -4.8 | -4.67 | -5 | -4.96 | -4.42 | -3.28 | -2.63 | -4.3 | -4.09 | -5.76 | -4 | -4.27 | -3.71 | -4.02 |
| *OsATL3* | Os02g49510 | -3.69 | -3.26 | -4.06 | -3.83 | -4.61 | -4 | -4.36 | -2.96 | -3.28 | -2.96 | -3.66 | -3.78 | -4.45 | -3.76 | -3.15 | -4.1 | -3.76 | -4.39 | -4.04 |
| *OsAAP12* | Os12g09320 | -3.32 | -3.39 | -4.36 | -3.8 | -3.75 | -4.1 | -3.77 | -3.02 | -3.13 | -3.24 | -3.31 | -4.37 | -4.01 | -2.89 | -2.44 | -3.5 | -4.12 | -3.62 | -3.66 |
| *OsATL16* | Os01g41400 | -3.87 | -3.22 | -3.08 | -3.4 | -4.17 | -3.2 | -3.67 | -4 | -3.52 | -2.89 | -3.97 | -4.1 | -3.41 | -2.92 | -2.65 | -3.6 | -3.96 | -3.84 | -3.64 |
| *OsATL14* | Os04g38660 | -5.3 | -5 | -4.79 | -5.14 | -5.51 | -5 | -4.89 | -5.66 | -5.75 | -5.63 | -5.55 | -6 | -5.26 | -4.54 | -3.58 | -6.1 | -5.11 | -5.42 | -5.47 |
| *OsATL17* | Os01g40410 | -3.03 | -2.46 | -2.72 | -3.28 | -3.12 | -3.3 | -3.24 | -2.86 | -3.18 | -2.81 | -2.68 | -3.29 | -2.87 | -1.97 | -1.38 | -3 | -2.81 | -2.66 | -3.23 |
| *OsATL8* | Os11g19240 | -2.8 | -3.19 | -3.96 | -2.98 | -4 | -3.4 | -3.12 | -3.03 | -2.93 | -4.1 | -3.17 | -3.26 | -2.77 | -2.13 | -1.77 | -3.1 | -3.13 | -3.62 | -3.26 |
| *OsBAT3* | Os01g71710 | -1.71 | -3.04 | -3.24 | -1.73 | -2.76 | -2.9 | -3.39 | -2.33 | -2.75 | -2.74 | -3.13 | -3.08 | -2.41 | -2.01 | -1.9 | -2.5 | -3.23 | -3.04 | -3.12 |
| *OsAAP3* | Os06g36180 | -5.17 | -5.09 | -4.76 | -5.08 | -4.68 | -5 | -4.63 | -4.95 | -5.94 | -4.77 | -5.36 | -3.43 | -1.55 | -1.73 | -4.18 | -5.2 | -4.84 | -4.52 | -5.12 |
| *OsLAT3* | Os03g25869 | -5.04 | -3.31 | -4.34 | -3.57 | -3.2 | -3.8 | -2.9 | -2.79 | -2.7 | -3.61 | -1.62 | -1.85 | -2.35 | -1.46 | -1.46 | -2.6 | -2.82 | -2.6 | -2.89 |
| *OsANT2* | Os03g60260 | -2.54 | -3.19 | -2.69 | -4 | -3.01 | -3.6 | -2.64 | -1.84 | -2.16 | -1.53 | -1.72 | -1.61 | -1.69 | -1.69 | -2.09 | -2.5 | -1.25 | -1.32 | -2.24 |
| *OsGAT2* | Os01g43320 | -4.21 | -4.32 | -3.46 | -4.62 | -4.36 | -3.9 | -3.23 | -2.33 | -2.01 | -4.12 | -2.69 | -1.17 | -1.32 | -1.5 | -3.17 | -3.2 | -1.45 | -2.08 | -3.48 |
| *OsLAT7* | Os12g39080 | 0.61 | -0.06 | -1.99 | -2.49 | -2.15 | -2.6 | -1.69 | -1.09 | -0.1 | -0.07 | 0 | 0.31 | 0.98 | 1.11 | 0.33 | -0.5 | -1.36 | -1.18 | -0.77 |
| *OsAAP6* | Os01g65670 | -0.07 | -2.71 | -2.21 | -3.83 | -3.05 | -3.2 | -2.39 | -2.85 | -2.97 | -1.67 | -0.31 | -0.29 | 1.44 | 2.76 | 1.36 | -0.4 | 1.13 | 0.3 | 0.09 |
| *OsProT2* | Os03g44230 | 0.02 | 0.74 | -0.18 | -0.47 | -1.06 | -1.1 | -1.07 | -1.48 | -0.8 | -0.23 | -0.45 | 0.02 | 0.51 | 0.36 | -0.5 | 0.29 | -0.2 | 0.01 | -0.37 |
| *OsCAT1* | Os01g11160 | -1.05 | 1.25 | -1.13 | -2.92 | -3.06 | -3.2 | -2.65 | -2.17 | -1.54 | -1.49 | 0.61 | 0.46 | 0.36 | 0.05 | -1.18 | -0.6 | -1.68 | -0.94 | -1.41 |
| *OsAAP13* | Os04g39489 | 0.45 | -1.12 | -1.65 | -4.15 | -3.72 | -4 | -2.87 | -1.79 | -1.45 | -1.02 | -0.27 | -0.06 | 0.16 | -0.62 | -2.98 | -0.6 | 0.38 | -0.12 | -1.11 |
| *OsAAP11* | Os11g09020 | -1.8 | -0.05 | -0.15 | -2.44 | -0.67 | -2.1 | -1.29 | -1.44 | -1.26 | -0.62 | 0.99 | 1.24 | 0.96 | 0.33 | -3.5 | -1 | 1.96 | 1.58 | -1.37 |
| *OsBAT7* | Os04g35540 | 0.25 | 0.57 | -1.2 | -3.65 | 0.06 | -1.4 | 0.67 | 0 | -1.75 | 1.32 | 0.8 | -0.27 | -0.48 | -0.57 | -0.56 | -0.4 | -0.23 | 0.36 | -1.49 |
| *OsANT4* | Os04g47780 | 0.33 | 2.78 | 0.73 | -2.5 | 0.46 | -0.1 | -0.64 | -0.36 | -3.23 | 1.27 | 1.52 | 0.38 | -1.04 | -1.17 | -2.07 | 0.15 | -0.21 | 0.69 | 0.05 |
| *OsAUX4* | Os10g05690 | 0.85 | -4.32 | -3.6 | 1.31 | -0.47 | 0.15 | -1.07 | -1.98 | -2.3 | -4.57 | -4.12 | -4.15 | -2.88 | -2.7 | -1.93 | -1.7 | -2.6 | -2.47 | -2.84 |
| *OsLHT6* | Os12g30040 | -2.52 | -4.02 | -4.28 | 0.49 | -0.3 | -0.4 | -0.09 | -0.39 | -0.78 | -1.18 | -1.96 | -2.36 | -1.57 | -2.08 | -2.82 | -1.7 | -1.75 | -2 | -2.03 |
| *OsATL13* | Os04g38680 | -0.68 | -2.78 | -1.06 | -0.38 | 0.29 | 1.59 | 0.87 | 0.98 | 0.21 | 0.31 | -1.22 | -1.32 | -1.84 | 0.53 | 1.56 | 0.41 | 1.87 | 1.85 | -0.02 |
| *OsANT1* | Os07g12770 | -2.64 | -0.48 | -0.47 | -0.78 | -0.56 | 0 | 0.03 | -0.28 | -1.19 | -0.8 | -0.25 | 0.06 | -0.35 | 0.09 | 0.77 | -1.4 | -2.29 | -1.68 | -1.45 |

YR, roots of 7-day-old seedlings; ML, mature leaves, YL, leaves of 7-day-old seedlings; SAM, up to 0.5 mm, shoot apical meristem; P1, 0-3 cm panicles; P2, 3-5 cm panicles; P3, 5-10 cm panicles; P4, 10-15 cm panicles; P5, 15-22 cm panicles; P6, 22-30 cm panicles; S1, 0-2 DAP seeds; S2, 3-4 DAP seeds; S3, 5-10 DAP seeds; S4, 11-20 DAP seeds;S5, 21-29 DAP seeds. CK, control; DS, drought stress; SS, salt stress; CS, cold stress.
