# Supplementary material for: Genome-Wide Survey and Expression Analysis of Amino Acid Transporter Gene Family in Rice (Oryza sativa L.)
Source: PLoS One. 2012 Nov 15;7(11):e49210. doi: 10.1371/journal.pone.0049210 (PMC3499563; doi:10.1371/journal.pone.0049210)
Supplement: Table S5 — The MPSS analysis of OsAAT genes. (DOC) [file pone.0049210.s010.doc]

**Table S5. The MPSS analysis of *OsAAT*** genes.

| **Gene** | **Locus** | **NYR** | **NST** | **NYL** | **NME** | **NPO** | **NOS** | **NIP** | **NCA** | **Max** |
| --- | --- | --- | --- | --- | --- | --- | --- | --- | --- | --- |
| *OsAAP1* | LOC_Os07g04180 | 0 | 8 | 336 | 2 | 0 | 63 | 4 | 78 | 336 |
| *OsAAP2* | LOC_Os06g12330 | 0 | 0 | 0 | 0 | 0 | 0 | 0 | 0 | 0 |
| *OsAAP3* | LOC_Os06g36180 | 264 | 118 | 135 | 124 | 26 | 115 | 62 | 141 | 264 |
| *OsAAP4* | LOC_Os12g09300 | 0 | 0 | 26 | 6 | 0 | 0 | 16 | 0 | 26 |
| *OsAAP5* | LOC_Os01g65660 | 57 | 41 | 7 | 107 | 0 | 0 | 17 | 0 | 107 |
| *OsAAP6* | LOC_Os01g65670 | 22 | 7 | 0 | 37 | 0 | 10 | 3 | 185 | 185 |
| *OsAAP7* | LOC_Os05g34980 | 2 | 0 | 0 | 0 | 0 | 0 | 0 | 0 | 2 |
| *OsAAP8* | LOC_Os01g66010 | 124 | 48 | 42 | 386 | 0 | 0 | 46 | 0 | 386 |
| *OsAAP9* | LOC_Os02g01210 | 36 | 60 | 38 | 24 | 0 | 0 | 33 | 0 | 60 |
| *OsAAP10* | LOC_Os02g49060 | 2 | 0 | 0 | 0 | 0 | 0 | 0 | 0 | 2 |
| *OsAAP11* | LOC_Os11g09020 | 0 | 0 | 220 | 0 | 0 | 83 | 78 | 0 | 220 |
| *OsAAP12* | LOC_Os12g09320 | 0 | 0 | 0 | 0 | 0 | 0 | 0 | 0 | 0 |
| *OsAAP13* | LOC_Os04g39489 | 90 | 99 | 110 | 3 | 370 | 1 | 0 | 21 | 370 |
| *OsAAP14* | LOC_Os04g56470 | 1 | 64 | 0 | 47 | 0 | 0 | 16 | 84 | 84 |
| *OsAAP15* | LOC_Os12g08130 | 0 | 0 | 0 | 0 | 1 | 0 | 5 | 10 | 5 |
| *OsAAP16* | LOC_Os12g08090 | 0 | 10 | 0 | 63 | 19 | 6 | 0 | 0 | 63 |
| *OsAAP17* | LOC_Os06g12350 | 0 | 0 | 0 | 0 | 0 | 0 | 0 | 0 | 0 |
| *OsAAP18* | LOC_Os06g36210 | 0 | 0 | 0 | 0 | 281 | 0 | 0 | 79 | 281 |
| *OsAAP19* | LOC_Os04g41350 | 0 | 48 | 0 | 142 | 0 | 0 | 0 | 0 | 142 |
| *OsLHT1* | LOC_Os04g47420 | 0 | 0 | 0 | 0 | 0 | 0 | 0 | 13 | 13 |
| *OsLHT2* | LOC_Os04g38860 | 0 | 0 | 0 | 0 | 0 | 0 | 0 | 0 | 0 |
| *OsLHT3* | LOC_Os05g14820 | 0 | 0 | 0 | 0 | 1 | 0 | 0 | 0 | 7 |
| *OsLHT4* | LOC_Os08g03350 | 1 | 14 | 22 | 0 | 8 | 4 | 0 | 5 | 22 |
| *OsLHT5* | LOC_Os12g14100 | - | - | - | - | - | - | - | - | - |
| *OsLHT6* | LOC_Os12g30040 | 0 | 0 | 0 | 8 | 0 | 0 | 25 | 46 | 46 |
| *OsGAT1* | LOC_Os05g50920 | 0 | 0 | 0 | 0 | 0 | 0 | 0 | 0 | 0 |
| *OsGAT2* | LOC_Os01g43320 | 0 | 0 | 0 | 0 | 0 | 0 | 0 | 0 | 0 |
| *OsGAT3* | LOC_Os10g27980 | 0 | 0 | 0 | 0 | 0 | 0 | 0 | 5 | 0 |
| *OsGAT4* | LOC_Os01g63854 | 124 | 6 | 197 | 0 | 76 | 67 | 22 | 40 | 197 |
| *OsProT1* | LOC_Os01g68050 | 0 | 0 | 0 | 1 | 0 | 0 | 0 | 0 | 1 |
| *OsProT2* | LOC_Os03g44230 | 16 | 0 | 21 | 14 | 33 | 24 | 0 | 5 | 33 |
| *OsProT3* | LOC_Os07g01090 | 0 | 0 | 42 | 7 | 0 | 0 | 5 | 0 | 42 |
| *OsAUX1* | LOC_Os01g63770 | 58 | 490 | 62 | 674 | 15 | 304 | 284 | 126 | 674 |
| *OsAUX2* | LOC_Os05g37470 | 0 | 0 | 0 | 2 | 0 | 0 | 0 | 0 | 2 |
| *OsAUX3* | LOC_Os03g14080 | 0 | 0 | 0 | 7 | 0 | 0 | 0 | 13 | 13 |
| *OsAUX4* | LOC_Os10g05690 | 0 | 4 | 0 | 0 | 0 | 2 | 0 | **95** | 95 |
| *OsAUX5* | LOC_Os11g06820 | 3 | 0 | 0 | 0 | 0 | 0 | 0 | 0 | 3 |
| *OsANT1* | LOC_Os07g12770 | 0 | 0 | 2 | 0 | 36 | 119 | 32 | 42 | 119 |
| *OsANT2* | LOC_Os03g60260 | 0 | 0 | 0 | 65 | 0 | 0 | 23 | 0 | 65 |
| *OsANT3* | LOC_Os02g44980 | 13 | 0 | 125 | 3 | 0 | 83 | 92 | 15 | 125 |
| *OsANT4* | LOC_Os04g47780 | 158 | 88 | 180 | 185 | 41 | 0 | 0 | 0 | 185 |
| *OsATL1* | LOC_Os06g43700 | 0 | 0 | 1 | 0 | 12 | 3 | 55 | 0 | 55 |
| *OsATL2* | LOC_Os09g26290 | 0 | 0 | 0 | 0 | 0 | 0 | 0 | 0 | 0 |
| *OsATL3* | LOC_Os02g49510 | 0 | 0 | 0 | 0 | **179** | 3 | 0 | 0 | 179 |
| *OsATL4* | LOC_Os06g16420 | 0 | 0 | 1 | 0 | 0 | 0 | 0 | 0 | 1 |
| *OsATL5* | LOC_Os06g42720 | 962 | 503 | 365 | 1362 | 236 | 238 | 712 | 378 | 1362 |
| *OsATL6* | LOC_Os02g09810 | 18 | 0 | 9 | 0 | 1 | 0 | 3 | 0 | 18 |
| *OsATL7* | LOC_Os01g61044 | 0 | 0 | 0 | 0 | 0 | **23** | 0 | 3 | 23 |
| *OsATL8* | LOC_Os11g19240 | 0 | 0 | 4 | 0 | 0 | 0 | 0 | 0 | 4 |
| *OsATL9* | LOC_Os02g54730 | 0 | 0 | 0 | 0 | 0 | 11 | 1 | 7 | 11 |
| *OsATL10* | LOC_Os12g38570 | 0 | 0 | 0 | 0 | **204** | 0 | 0 | 0 | 204 |
| *OsATL11* | LOC_Os02g01100 | 1 | 0 | 12 | 2 | 0 | 27 | 0 | 60 | 60 |
| *OsATL12* | LOC_Os06g12320 | 0 | 0 | **18** | 3 | 0 | 0 | 0 | 0 | 18 |
| *OsATL13* | LOC_Os04g38680 | 0 | 0 | 0 | 0 | 0 | 19 | 15 | 0 | 19 |
| *OsATL14* | LOC_Os04g38660 | - | - | - | - | - | - | - | - | - |
| *OsATL15* | LOC_Os01g41420 | 472 | 689 | 168 | 296 | 29 | 31 | 0 | 0 | 689 |
| *OsATL16* | LOC_Os01g41400 | 0 | 0 | 0 | 0 | **60** | 0 | 0 | 0 | 60 |
| *OsATL17* | LOC_Os01g40410 | - | - | - | - | - | - | - | - | - |
| *OsCAT1* | LOC_Os01g11160 | 0 | 0 | 15 | 0 | 0 | 4 | 0 | 0 | 15 |
| *OsCAT2* | LOC_Os02g43860 | 16 | 35 | 120 | 240 | 166 | 29 | 96 | 116 | 240 |
| *OsCAT3* | LOC_Os03g43970 | - | - | - | - | - | - | - | - | - |
| *OsCAT4* | LOC_Os03g45170 | 112 | 11 | 79 | 88 | 58 | 0 | 9 | 5 | 112 |
| *OsCAT5* | LOC_Os04g45950 | 0 | 0 | 0 | 0 | 0 | 0 | 0 | 0 | 0 |
| *OsCAT6* | LOC_Os06g34830 | 0 | 1 | 0 | 25 | 2 | 0 | 59 | 0 | 59 |
| *OsCAT7* | LOC_Os10g30090 | 0 | 5 | 2 | 0 | 24 | 0 | 0 | 0 | 24 |
| *OsCAT8* | LOC_Os11g05690 | 0 | 0 | 0 | 0 | 0 | 0 | 0 | 0 | 0 |
| *OsCAT9* | LOC_Os12g06060 | 0 | 0 | 0 | 0 | 0 | 7 | 25 | 0 | 25 |
| *OsCAT10* | LOC_Os12g41890 | 0 | 0 | 0 | 0 | 0 | 0 | 0 | 0 | 0 |
| *OsCAT11* | LOC_Os12g42850 | 249 | 87 | 347 | 129 | 0 | 44 | 66 | 72 | 347 |
| *OsBAT1* | LOC_Os01g42234 | 53 | 8 | **110** | 43 | 15 | 5 | 36 | 25 | 110 |
| *OsBAT2* | LOC_Os01g71700 | 0 | 0 | 0 | 0 | 0 | 0 | 0 | 0 | 0 |
| *OsBAT3* | LOC_Os01g71710 | 0 | 0 | 0 | 0 | 0 | 0 | 0 | 0 | 0 |
| *OsBAT4* | LOC_Os01g71720 | 0 | 0 | **85** | 0 | 0 | 0 | 0 | 0 | 85 |
| *OsBAT5* | LOC_Os01g71740 | 0 | 0 | 0 | 0 | 0 | 0 | 0 | 0 | 0 |
| *OsBAT6* | LOC_Os01g71760 | 0 | 0 | 0 | 0 | 0 | 0 | 0 | 0 | 0 |
| *OsBAT7* | LOC_Os04g35540 | 0 | 0 | 0 | 0 | 0 | 36 | 0 | 13 | 36 |
| *OsLAT1* | LOC_Os02g47210 | 10 | 0 | 2 | 2 | 0 | 6 | 14 | 8 | 14 |
| *OsLAT2* | LOC_Os03g25840 | 0 | 0 | 0 | 0 | 0 | 0 | 0 | 0 | 0 |
| *OsLAT3* | LOC_Os03g25869 | 0 | 0 | 0 | 0 | 0 | 0 | 0 | 0 | 0 |
| *OsLAT4* | LOC_Os03g25920 | 19 | 0 | 0 | 0 | 26 | 0 | 13 | 0 | 26 |
| *OsLAT5* | LOC_Os03g37984 | 28 | 45 | 11 | 0 | 0 | 0 | 0 | 18 | 45 |
| *OsLAT6* | LOC_Os08g41370 | - | - | - | - | - | - | - | - | - |
| *OsLAT7* | LOC_Os12g39080 | 0 | 0 | 7 | 0 | 0 | 0 | 0 | 0 | 7 |
| *OsLAT8* | LOC_Os01g19850 | 0 | 0 | 0 | 0 | 3 | 0 | 0 | 0 | 3 |
| *OsLAT9* | LOC_Os08g23440 | 62 | 113 | 16 | 323 | 50 | 58 | 58 | 74 | 323 |

Underlined and bold indicated specific expression; underlined indicated abundant expression; –, no expressed signatures. NYR, 14 d young roots; NST, 60 d stem; NYL, 14 d young leaves; NME, 60 d meristem tissue; NPO, mature pollen; NOS, ovary and mature stigma; NIP, 90 d immature panicle; NCA, 35 d callus; Max, maximum value.
