# Supplementary material for: Genome-Wide Survey and Expression Analysis of Amino Acid Transporter Gene Family in Rice (Oryza sativa L.)
Source: PLoS One. 2012 Nov 15;7(11):e49210. doi: 10.1371/journal.pone.0049210 (PMC3499563; doi:10.1371/journal.pone.0049210)
Supplement: Table S6 — Data for expression comparison of OsAAT and AtAAT genes. (DOC) [file pone.0049210.s011.doc]

**Table S6. Data for expession comparison of *OsAATs* and *AtAATs***.

| **Gene** | **R** | **L** | **I** | **P** | **S** | **DSS** | **DSR** | **SSS** | **SSR** | **CSS** | **CSR** |
| --- | --- | --- | --- | --- | --- | --- | --- | --- | --- | --- | --- |
| *OsAAP1* | 3.21 | 1.19 | 1.55 | 0 | 7.34 | 1.06 | | 1.19 | | 0.60 | |
| *AtAAP1* | 0.57 | 1.78 | 11.2 | 0.07 | 6.29 | 2.24 | 1.07 | 1.49 | 3.74 | 0.93 | 1.09 |
| *AtAAP8* | 0.05 | 0.07 | 0.34 | 0.07 | 0.25 | 1.11 | 0.30 | 1.14 | 0.48 | 1.66 | 0.33 |
| *AtAAP6* | 0.81 | 0.68 | 1.66 | 0.11 | 0.80 | 1.37 | 0.83 | 0.83 | 1.75 | 0.78 | 1.82 |
| *OsAAP3* | 0.03 | 0.04 | 0.04 | 26 | 0.35 | 1.31 | | 1.63 | | 1.08 | |
| *OsAAP2* | 0.02 | 0.04 | 0.4 | 0 | 0.06 | 1.30 | | 0.82 | | 0.93 | |
| *OsAAP18* | 0.24 | 0.06 | 0.62 | 281 | 0.45 | 1.63 | | 1.68 | | 1.37 | |
| *OsAAP11* | 0.29 | 0.99 | 0.67 | 0 | 2.43 | 7.92 | | 6.08 | | 0.79 | |
| *OsAAP15* | 0.52 | 3.93 | 9.36 | 1.00 | 10.20 | 2.25 | | 2.04 | | 2.12 | |
| *OsAAP16* | 2.13 | 6.8 | 13.4 | 19 | 14.50 | 1.43 | | 1.06 | | 0.66 | |
| *OsAAP12* | 0.10 | 0.10 | 0.13 | 0 | 0.19 | 0.63 | | 0.89 | | 0.87 | |
| *OsAAP19* | 0.09 | 0.06 | 0.05 | 0 | 0.17 | 0.81 | | 1.20 | | 0.97 | |
| *AtAAP2* | 7.14 | 0.64 | 2.94 | 0.35 | 7.07 | 1.00 | 0.96 | 1.23 | 0.77 | 0.78 | 1.10 |
| *AtAAP4* | 1.05 | 0.29 | 12.2 | 0.26 | 1.12 | 2.12 | 0.94 | 0.61 | 2.36 | 1.04 | 1.53 |
| *AtAAP3* | 0.38 | 0.02 | 2.18 | 0.02 | 1.45 | 1.16 | 1.30 | 0.47 | 2.16 | 0.91 | 0.61 |
| *AtAAP5* | 0.95 | 0.32 | 7.87 | 3.11 | 1.80 | 1.15 | 0.86 | 0.96 | 3.63 | 1.86 | 1.24 |
| *OsAAP7* | 1.84 | 2.36 | 1.07 | 0 | 0.68 | 0.77 | | 0.63 | | 0.79 | |
| *OsAAP8* | 0.80 | 0.66 | 0.35 | 0 | 0.31 | 0.33 | | 0.42 | | 0.51 | |
| *OsAAP9* | 0.08 | 0.52 | 0.08 | 0 | 0.13 | 0.29 | | 0.59 | | 0.75 | |
| *OsAAP6* | 0.98 | 0.22 | 0.32 | 0 | 6.95 | 2.80 | | 1.57 | | 1.36 | |
| *OsAAP5* | 0.65 | 0.42 | 0.52 | 0 | 0.26 | 1.67 | | 2.52 | | 0.89 | |
| *OsAAP4* | 2.92 | 0.50 | 0.52 | 0 | 0.37 | 0.18 | | 0.37 | | 0.53 | |
| *OsAAP17* | 0.02 | 0.04 | 1.2 | 0 | 0.04 | 1.33 | | 1.09 | | 1.19 | |
| *AtAAP7* | 0.15 | 0.16 | 0.62 | 0.20 | 0.27 | 1.38 | 1.68 | 0.68 | 2.04 | 0.44 | 1.06 |
| *OsAAP10* | 0.44 | 0.12 | 0.18 | 0 | 0.33 | 0.59 | | 0.57 | | 0.85 | |
| *OsAAP13* | 1.4 | 0.47 | 0.5 | 370 | 1.14 | 2.03 | | 1.43 | | 0.72 | |
| *OsAAP14* | 0.33 | 0.10 | 1.79 | 0 | 0.69 | 2.08 | | 2.68 | | 1.15 | |
| *AtLHT8* | 0.01 | 0.01 | 2.11 | 3.37 | 0.08 | 1.05 | 0.82 | 1.35 | 0.54 | 0.71 | 0.45 |
| *OsLHT1* | 10.8 | 11.2 | 0.66 | 0 | 15.7 | 1.02 | | 1.21 | | 1.08 | |
| *AtLHT1* | 3.85 | 0.38 | 10.2 | 0.29 | 1.7 | 1 | 0.65 | 1.09 | 1.17 | 0.77 | 0.89 |
| *AtLHT10* | 0.10 | 0.03 | 0.07 | 0.11 | 0.14 | 0.81 | 1.08 | 0.46 | 0.66 | 0.26 | 0.92 |
| *OsLHT2* | 0.06 | 0.06 | 0.12 | 0 | 0.14 | 0.76 | | 1.03 | | 0.77 | |
| *AtLHT2* | 0.04 | 0.37 | 2.57 | 6.55 | 0.38 | 0.96 | 0.9 | 0.85 | 1.39 | 0.46 | 1.56 |
| *AtLHT5* | 0.07 | 0.09 | 0.25 | 8.19 | 0.32 | 1.04 | 0.85 | 1.67 | 0.82 | 1.17 | 1.21 |
| *AtLHT6* | 0.07 | 0.01 | 0.03 | 0.04 | 0.03 | 7.82 | 1.74 | 2.64 | 0.64 | 2.11 | 1.42 |
| *AtLHT3* | 0.07 | 0.01 | 0.03 | 0.04 | 0.03 | 7.82 | 1.74 | 2.64 | 0.64 | 2.11 | 1.42 |
| *OsLHT3* | 0.03 | 0.05 | 0.31 | 1.00 | 0.46 | 3.68 | | 3.44 | | 1.39 | |
| *OsLHT4* | 1.29 | 0.1 | 14.1 | 8 | 0.24 | 0.65 | | 0.64 | | 0.84 | |
| *AtLHT9* | 1.41 | 0.15 | 0.91 | 0.04 | 7.00 | 0.73 | 0.71 | 1.36 | 0.92 | 0.51 | 0.82 |
| *OsLHT5* | 0.45 | 0.93 | 1.94 | - | 0.85 | 0.27 | | 0.45 | | 0.76 | |
| *OsLHT6* | 0.18 | 0.06 | 1.44 | 0 | 0.35 | 0.98 | | 0.82 | | 0.81 | |
| *AtLHT4* | 1.91 | 0.42 | 1.02 | 0.03 | 0.67 | 0.78 | 0.91 | 0.65 | 0.48 | 0.68 | 1.26 |
| *AtLHT7* | 0.13 | 0.05 | 10.8 | 74.9 | 1.67 | 0.84 | 0.78 | 1.31 | 11.30 | 1.14 | 0.44 |
| *OsProT1* | 0.10 | 0.05 | 0.10 | 0 | 0.06 | 0.35 | | 0.52 | | 0.97 | |
| *AtProT1* | 1.32 | 0.35 | 9.18 | 33.6 | 1.86 | 0.97 | 1.00 | 0.68 | 1.29 | 0.86 | 1.18 |
| *AtProT3* | 0.09 | 0.36 | 2.21 | 0.30 | 0.22 | 1.78 | 1.11 | 1.83 | 0.94 | 1.14 | 0.86 |
| *AtProT2* | 0.2 | 0.18 | 0.81 | 0.05 | 0.35 | 0.98 | 1.00 | 0.91 | 4.23 | 0.94 | 0.87 |
| *OsProT2* | 1.04 | 1.72 | 0.88 | 33 | 1.46 | 0.71 | | 0.82 | | 0.63 | |
| *OsProT3* | 1.35 | 0.42 | 0.20 | 0 | 0.15 | 0.24 | | 0.49 | | 0.78 | |
| *OsGAT4* | 0.99 | 0.33 | 0.42 | 76 | 0.49 | 1.07 | | 1.12 | | 0.84 | |
| *OsGAT2* | 0.06 | 0.09 | 0.25 | 0 | 0.46 | 3.44 | | 2.22 | | 0.84 | |
| *OsGAT1* | 0.52 | 0.05 | 0.18 | 0 | 1.09 | 1.05 | | 1.22 | | 2.39 | |
| *AtGATL1* | 0.26 | 0.33 | 1.07 | 0.31 | 1.29 | 1.16 | 1.15 | 1.12 | 1.58 | 0.78 | 0.89 |
| *OsGAT3* | 1.67 | 0.22 | 0.07 | 0 | 0.28 | 0.31 | | 0.23 | | 0.76 | |
| *AtGAT1* | 0.15 | 0.17 | 1.19 | 0.09 | 0.56 | 1.3 | 1.14 | 2.67 | 1.58 | 0.92 | 1.19 |
| *AtLAX1* | 0.83 | 1.34 | 0.93 | 0.09 | 0.63 | 0.66 | 0.92 | 0.89 | 0.46 | 0.81 | 1.32 |
| *AtAUX1* | 4.71 | 2.48 | 8.58 | 0.13 | 1.61 | 0.8 | 1.06 | 0.33 | 0.91 | 0.9 | 1.08 |
| *OsAUX1* | 5.56 | 1.78 | 8.25 | 15 | 2.21 | 0.57 | | 0.59 | | 0.53 | |
| *OsAUX2* | 1.96 | 0.67 | 1.62 | 0 | 1.35 | 0.36 | | 0.41 | | 0.75 | |
| *AtLAX2* | 1.15 | 0.68 | 1.76 | 0.07 | 1.17 | 0.87 | 0.97 | 0.6 | 0.46 | 0.89 | 1.51 |
| *AtLAX3* | 4.42 | 0.23 | 2.42 | 0.09 | 0.53 | 0.91 | 0.82 | 0.40 | 0.50 | 0.82 | 1.58 |
| *OsAUX3* | 0.13 | 0.08 | 1.19 | 0 | 0.08 | 0.77 | | 0.90 | | 0.88 | |
| *OsAUX4* | 1.85 | 0.08 | 2.55 | 0 | 0.27 | 0.53 | | 0.58 | | 0.45 | |
| *OsAUX5* | 0.68 | 0.11 | 0.88 | 0 | 0.13 | 1.23 | | 0.97 | | 0.45 | |
| *AtANT1* | 0.74 | 0.90 | 2.29 | 9.48 | 1.61 | 1.13 | 1.07 | 1.07 | 1.05 | 1.22 | 0.84 |
| *OsANT1* | 0.16 | 0.74 | 1.05 | 36 | 1.75 | 0.55 | | 0.85 | | 0.99 | |
| *OsANT2* | 0.18 | 0.16 | 0.35 | 0 | 0.34 | 2.38 | | 2.26 | | 1.2 | |
| *AtANT2* | 0.16 | 1.07 | 1.68 | 0.27 | 1.61 | 1.09 | 1.54 | 2.61 | 14.2 | 0.81 | 1.56 |
| *AtANT4* | **×** | **×** | **×** | **×** | **×** | **×** | **×** | **×** | **×** | **×** | **×** |
| *AtANT3* | 2.38 | 2.2 | 5.94 | 5.75 | 3.75 | 1.29 | 0.96 | 1.11 | 1.07 | 0.77 | 1.07 |
| *OsANT3* | 2.26 | 4.44 | 6.53 | 0 | 10.4 | 3.60 | | 3.41 | | 3.77 | |
| *OsANT4* | 1.29 | 7.02 | 2.47 | 41 | 2.95 | 0.78 | | 1.45 | | 0.93 | |
| *AtVAAT10* | **×** | **×** | **×** | **×** | **×** | **×** | **×** | **×** | **×** | **×** | **×** |
| *OsATL8* | 0.15 | 0.11 | 0.13 | 0 | 0.30 | 0.96 | | 0.68 | | 0.87 | |
| *AtVAAT3* | 0.38 | 0.25 | 1.37 | 0.65 | 1.15 | 1.22 | 1.01 | 37.6 | 3.01 | 0.88 | 0.95 |
| *OsATL9* | 1.58 | 4.66 | 2.23 | 0 | 2.33 | 0.37 | | 0.71 | | 0.79 | |
| *AtVAAT9* | 0.12 | 0.32 | 0.96 | 5.90 | 0.39 | 1.17 | 1.10 | 0.99 | 1.1 | 0.83 | 0.94 |
| *AtVAAT8* | 0.41 | 0.14 | 0.57 | 0.48 | 0.21 | 1.18 | 1.31 | 1.05 | 3.51 | 1.24 | 1.05 |
| *AtVAAT7* | 0.18 | 0.08 | 0.09 | 0.07 | 0.22 | 0.79 | 0.97 | 1.50 | 0.63 | 0.94 | 0.92 |
| *AtVAAT6* | 0.18 | 0.08 | 0.09 | 0.07 | 0.22 | 0.79 | 0.97 | 1.50 | 0.63 | 0.94 | 0.92 |
| *OsATL11* | 1.05 | 1.02 | 0.64 | 0 | 0.65 | 1.15 | | 2.03 | | 0.97 | |
| *AtVAAT5* | **×** | **×** | **×** | **×** | **×** | **×** | **×** | **×** | **×** | **×** | **×** |
| *AtVAAT4* | 0.84 | 0.38 | 1.98 | 2.73 | 0.29 | 1.27 | 0.92 | 0.89 | 0.71 | 1.19 | 0.92 |
| *OsATL10* | 0.06 | 0.06 | 0.52 | 204 | 0.11 | 0.85 | | 1.07 | | 1.00 | |
| *AtVAAT2* | 0.17 | 0.19 | 0.64 | 0.32 | 1.48 | 0.68 | 1.16 | 0.86 | 2.46 | 3.99 | 1.87 |
| *AtVAAT1* | 0.08 | 0.07 | 0.46 | 0.11 | 0.52 | 0.86 | 1.30 | 0.76 | 1.49 | 0.97 | 1.11 |
| *OsATL13* | 0.64 | 0.49 | 3.10 | 0 | 3.03 | 2.76 | | 2.72 | | 0.74 | |
| *OsATL14* | 0.03 | 0.04 | 0.03 | - | 0.09 | 2.02 | | 1.62 | | 1.57 | |
| *OsATL12* | 2.00 | 0.43 | 5.19 | 0 | 0.11 | 0.50 | | 0.53 | | 0.48 | |
| *OsATL15* | 0.09 | 0.37 | 0.11 | 29 | 0.24 | 1.60 | | 1.29 | | 1.62 | |
| *OsATL16* | 0.07 | 0.12 | 0.14 | 60 | 0.16 | 0.76 | | 0.83 | | 0.95 | |
| *OsATL17* | 0.13 | 0.19 | 0.15 | - | 0.39 | 1.16 | | 1.28 | | 0.87 | |
| *OsATL1* | 0.89 | 0.67 | 1.24 | 12 | 0.78 | 0.96 | | 1.08 | | 0.37 | |
| *AtT5* | 0.49 | 0.48 | 0.81 | 2.21 | 0.70 | 0.97 | 1.14 | 0.91 | 1.07 | 0.85 | 1.33 |
| *AtT4* | 0.34 | 0.18 | 0.92 | 2.58 | 0.42 | 0.95 | 1.07 | 1.43 | 1.01 | 1.05 | 1.97 |
| *AtT3* | 6.91 | 3.11 | 9.53 | 5.63 | 5.25 | 1.16 | 1.02 | 1.08 | 0.90 | 0.76 | 1.05 |
| *OsATL5* | 13.7 | 13.1 | 15.5 | 236 | 10.9 | 0.84 | | 1.00 | | 1.12 | |
| *OsATL6* | 1.70 | 8.37 | 5.77 | 1 | 6.06 | 9.12 | | 8.39 | | 2.82 | |
| *OsATL4* | 1.96 | 4.4 | 3.07 | 0 | 2.85 | 1.14 | | 1.24 | | 1.08 | |
| *OsATL3* | 0.08 | 0.11 | 0.13 | 179 | 0.12 | 1.24 | | 0.80 | | 1.02 | |
| *OsATL2* | 0.02 | 0.05 | 0.04 | 0 | 0.03 | 1.45 | | 1.49 | | 0.75 | |
| *AtT2* | 0.51 | 0.34 | 2.23 | 0.07 | 1.45 | 1.34 | 0.92 | 1.78 | 4.63 | 1.53 | 1.48 |
| *AtT1* | 0.76 | 0.50 | 1.58 | 4.05 | 1.01 | 0.87 | 1.14 | 0.87 | 1.12 | 0.62 | 0.87 |
| *OsATL7* | 0.02 | 0.04 | 0.03 | 0 | 0.02 | 1.16 | | 1.37 | | 0.98 | |
| *AtTTP1* | 0.09 | 0.13 | 0.11 | 0.36 | 0.17 | 1.07 | 0.52 | 1.54 | 0.73 | 1.11 | 1.01 |
| *AtTTP2* | 0.17 | 0.65 | 0.88 | 0.63 | 1.03 | 0.91 | 0.82 | 1.21 | 0.80 | 0.95 | 0.97 |
| *AtCAT1* | 0.15 | 0.11 | 2.64 | 1.15 | 0.79 | 1.01 | 1.19 | 0.48 | 1.63 | 1.29 | 0.90 |
| *OsCAT3* | **×** | **×** | **×** | **×** | **×** | **×** | | **×** | | **×** | |
| *OsCAT10* | **×** | **×** | **×** | 0 | **×** | **×** | | **×** | | **×** | |
| *OsCAT1* | 0.49 | 2.44 | 0.37 | 0 | 1.56 | 0.47 | | 0.79 | | 0.57 | |
| *AtCAT5* | 0.13 | 0.12 | 0.34 | 0.21 | 1.43 | 0.23 | 1.72 | 0.71 | 1.93 | 0.93 | 1.94 |
| *OsCAT6* | 0.12 | 0.12 | 0.7 | 2 | 0.68 | 2.62 | | 1.75 | | 0.73 | |
| *AtCAT8* | 1.01 | 0.92 | 1.28 | 0.13 | 0.95 | 0.91 | 1.00 | 0.99 | 1.04 | 0.67 | 1.01 |
| *OsCAT5* | 0.39 | 0.09 | 0.32 | 0 | 0.1 | 0.85 | | 0.85 | | 1.04 | |
| *AtCAT6* | 0.53 | 0.21 | 2.98 | 0.01 | 2.01 | 1.13 | 1.72 | 0.15 | 2.97 | 2.04 | 1.01 |
| *AtCAT7* | 0.16 | 0.13 | 0.16 | 0.34 | 0.12 | 0.59 | 0.95 | 0.72 | 0.92 | 0.81 | 1.34 |
| *OsCAT8* | **×** | **×** | **×** | 0 | **×** | **×** | | **×** | | **×** | |
| *OsCAT9* | 0.36 | 0.22 | 0.45 | 0 | 0.68 | 0.72 | | 0.57 | | 0.72 | |
| *AtCAT9* | 1.35 | 1.7 | 2.52 | 1.52 | 2.06 | 1.12 | 0.96 | 1 | 0.72 | 0.84 | 0.7 |
| *OsCAT2* | 1.32 | 1.56 | 0.88 | 166 | 1.01 | 0.88 | | 0.95 | | 0.97 | |
| *AtCAT2* | 2.05 | 1.84 | 8.7 | 42 | 2.5 | 1.21 | 1.07 | 1.31 | 0.88 | 0.81 | 1.02 |
| *AtCAT3* | 0.64 | 0.58 | 1.05 | 0.08 | 0.59 | 1.07 | 1.09 | 0.92 | 1.15 | 0.81 | 0.87 |
| *OsCAT11* | 8.83 | 16.3 | 7.62 | 0 | 9.29 | 1.53 | | 1.75 | | 1.2 | |
| *OsCAT4* | 1.31 | 1.44 | 0.84 | 58 | 0.32 | 0.94 | | 0.81 | | 0.99 | |
| *AtCAT4* | 0.53 | 0.75 | 2.43 | 7.24 | 1.35 | 1.17 | 0.88 | 1.14 | 1.01 | 0.91 | 0.71 |
| *OsCAT7* | 0.63 | 0.64 | 0.59 | 24 | 0.54 | 0.62 | | 0.65 | | 1.07 | |
| *OsBAT7* | 1.22 | 1.53 | 2.57 | 0 | 1.79 | 1.13 | | 1.70 | | 0.47 | |
| *AtBAT1* | 0.66 | 0.86 | 1.20 | 0.19 | 0.87 | 1.71 | 1.13 | 1.31 | 0.92 | 0.46 | 0.62 |
| *OsBAT1* | 1.02 | 0.51 | 0.39 | 15 | 0.73 | 1.37 | | 0.95 | | 0.89 | |
| *OsBAT2* | 0.04 | 0.09 | 0.05 | 0 | 0.06 | 0.6 | | 0.97 | | 0.48 | |
| *OsBAT3* | 0.31 | 0.12 | 0.31 | 0 | 0.27 | 0.61 | | 0.69 | | 0.66 | |
| *OsBAT4* | 0.06 | 6.31 | 0.06 | 0 | 0.13 | 0.11 | | 0.38 | | 0.36 | |
| *OsBAT5* | 0.04 | 0.13 | 0.07 | 0 | 0.08 | 1.23 | | 1.10 | | 0.85 | |
| *OsBAT6* | 0.15 | 0.2 | 0.24 | 0 | 0.12 | 1.88 | | 1.35 | | 1.50 | |
| *AtLAT1* | 0.09 | 0.24 | 0.46 | 0.04 | 0.16 | 0.80 | 1.14 | 0.71 | 0.55 | 0.64 | 0.75 |
| *AtLAT2* | 0.17 | 0.1 | 1.93 | 0.36 | 0.98 | 1.52 | 0.92 | 1.97 | 1.54 | 0.69 | 0.72 |
| *AtLAT3* | 0.62 | 0.56 | 1.25 | 1.36 | 3.36 | 1.35 | 0.86 | 1.49 | 0.5 | 0.77 | 0.84 |
| *OsLAT5* | 3.13 | 2.39 | 3.06 | 0 | 8.43 | 0.90 | | 0.89 | | 0.74 | |
| *OsLAT1* | 0.47 | 0.24 | 0.16 | 0 | 0.68 | 1.02 | | 1.13 | | 0.71 | |
| *OsLAT6* | **×** | **×** | **×** | **×** | **×** | **×** | | **×** | | **×** | |
| *AtLAT4* | 0.40 | 0.44 | 0.78 | 0.18 | 0.66 | 1.14 | 1.18 | 0.71 | 2.15 | 0.6 | 1.09 |
| *OsLAT4* | 0.74 | 0.33 | 0.79 | 26 | 0.9 | 0.81 | | 1.29 | | 0.41 | |
| *AtLAT5* | 0.29 | 0.45 | 0.62 | 0.21 | 0.53 | 1.36 | 1.17 | 2.62 | 1.44 | 1.13 | 0.69 |
| *OsLAT7* | 1.57 | 0.98 | 0.98 | 0 | 2.21 | 0.56 | | 0.64 | | 0.85 | |
| *OsLAT2* | **×** | **×** | **×** | 0 | **×** | **×** | | **×** | | **×** | |
| *OsLAT3* | 0.03 | 0.1 | 0.16 | 0 | 0.37 | 0.88 | | 1.02 | | 0.84 | |
| *OsLAT8* | 0.07 | 0.08 | 0.24 | 3 | 0.11 | 0.92 | | 1.08 | | 1.08 | |
| *OsLAT9* | 0.07 | 0.06 | 0.12 | 50 | 0.08 | 1.10 | | 1.09 | | 0.78 | |

R, root; L, leaf; I, inflorescence; P, pollen; S, silique or seed; DSS and DSR; drought stressed shoot and root; SSS and SSR, salt stressed shoot and root; CSS and CSR, cold stressed shoot and root. ×, no expressed signatures.
