# Supplementary material for: Genome-Wide Survey and Expression Analysis of Amino Acid Transporter Gene Family in Rice (Oryza sativa L.)
Source: PLoS One. 2012 Nov 15;7(11):e49210. doi: 10.1371/journal.pone.0049210 (PMC3499563; doi:10.1371/journal.pone.0049210)
Supplement: Table S7 — Correlation coefficients between expression patterns from homologous genes. (DOC) [file pone.0049210.s012.doc]

**Table S7. Correlation coefficients between expression patterns from homologous genes.**

| **Subfamilies** | **Homologous genes** | **Correlation coefficients** | **Note** |
| --- | --- | --- | --- |
| **LHT** | *AtLHT3* | 0.86 | Figure 7B |
|  | *OsLHT3* |  |  |
| **GAT** | *AtGATL1* | 0.50 | Figure 7C |
|  | *OsGAT1* |  |  |
| **AUX** | *AtAUX1* | 0.98 | Figure 7D |
|  | *OsAUX1* |  |  |
|  | *AtLAX2* | 0.84 | Figure 7E |
|  | *OsAUX2* |  |  |
| **ANT** | *AtANT2* | 0.69 | Figure 7F |
|  | *OsANT2* |  |  |
|  | *AtANT3* | 0.62 | Figure 7G |
|  | *OsANT3* |  |  |
| **ATLa** | *AtT5* | 0.93 | Figure 7H |
|  | *OsATL1* |  |  |
|  | *AtT3* | 0.77 | Figure 7I |
|  | *OsATL5* |  |  |
| **CAT** | *AtCAT5* | 0.19 | Figure 7J |
|  | *OsCAT1* |  |  |
| **ACT** | *AtBAT1* | 0.83 | Figure 7K |
|  | *OsBAT7* |  |  |
