# Supplementary material for: Genome-Wide Survey and Expression Analysis of Amino Acid Transporter Gene Family in Rice (Oryza sativa L.)
Source: PLoS One. 2012 Nov 15;7(11):e49210. doi: 10.1371/journal.pone.0049210 (PMC3499563; doi:10.1371/journal.pone.0049210)
Supplement: Table S8 — Primers used in qRT-PCR of OsAAT genes. (DOC) [file pone.0049210.s013.doc]

## Table S8. Primers used in qRT-PCR of rice *AAT* genes.

| **Genes** | **Primers used in qRT-PCR(5′→3′)** | |
| --- | --- | --- |
| *OsAAP3* | Forwards： | TCCTCTGCTTCCTCGTCT |
|  | Reverse： | CAAACTCTTGGCTAACTTCC |
| *OsAAP4* | Forwards： | AGCTCAGCAACAAGCAC |
|  | Reverse： | ACCAGCAGCAAGAAAAA |
| *OsAAP5* | Forwards： | GCCACTGACTGTTTACTTC |
|  | Reverse： | CCACTGCCACCGAGAT |
| *OsAAP6* | Forwards： | GTCACCATCTTCCAAACGC |
|  | Reverse： | ATACTCCTCAAGTCCCATT |
| *OsAAP7* | Forwards： | AGAGGAATTTGAGACAG |
|  | Reverse： | TAACATTAACACGGACC |
| *OsAAP8* | Forwards： | GCACCCAGAACGACAC |
|  | Reverse： | GACACGCACTTACACTTTTT |
| *OsAAP11* | Forwards： | TCAGCGGGTAGAGAGAA |
|  | Reverse： | CAAAACACCACACAAAG |
| *OsAAP13* | Forwards： | ATTTCGGATTTGCTTTTTCT |
|  | Reverse： | CACCACTATGCTATTGTTCA |
| *OsAAP15* | Forwards： | GTGCTGCCTCGTCGTCT |
|  | Reverse： | GGATTCTACTGCTCCCTTGT |
| *OsLHT6* | Forwards： | GTCACCTTCGCCTACCCGT |
|  | Reverse： | GCTCCAGACACCACCCACA |
| *OsGAT3* | Forwards： | TGTCCTCCCTGACTTTTCC |
|  | Reverse： | CTGTAGCCTGCGTCTTTCG |
| *OsProT1* | Forwards： | TCGTCAACCTCTTCGGCTC |
|  | Reverse： | CCATCTCCCTGCTTCATCC |
| *OsProT3* | Forwards： | TCTGTGACTGTGATTGA |
|  | Reverse： | AGTAGTAAGAGGTATGC |
| *OsANT1* | Forwards： | TGCGGGCTACGGGCTCATT |
|  | Reverse： | TGCCTTCGTTCCCACTTCC |
| *OsANT3* | Forwards： | ACGACAGATTCCCATAGC |
|  | Reverse： | AAGCTGCAACCTCAACTAG |
| *OsATL1* | Forwards： | ATTGTGATGCTTGGTTT |
|  | Reverse： | CCTCTGCTGTCTTCTGT |
| *OsATL6* | Forwards： | AGTAATACCCAGCAAAGCA |
|  | Reverse： | TGGCGGAATAGATACAAAC |
| *OsATL7* | Forwards： | GCAAAGACGAAGGACAAG |
|  | Reverse： | ACAGAGGAACGCTACAGG |
| *OsATL9* | Forwards： | AAAGCAGTAAACTTAGGTGGTA |
|  | Reverse： | CTCCGAACATGAGATAGCC |
| *OsATL12* | Forwards： | ATCATCGTGCTTGGGTTAG |
|  | Reverse： | ACGGCATATGGATTTCTTT |
| *OsATL13* | Forwards： | GCTCGGTTCGTTGGTTGC |
|  | Reverse： | CGGTGTTTCCATGCCTAAT |
| *OsATL15* | Forwards： | TGCAGATTATAGCCACT |
|  | Reverse： | CCCTTTATTTTAAGGAC |
| *OsAUX1* | Forwards： | GGTAGAAGAAGAAGAGGGC |
|  | Reverse： | CCAAACAAACACAAGGACA |
| *OsAUX2* | Forwards： | TGACTAGATTTACCGCCCTG |
|  | Reverse： | TTCACATCCAACTCCCAAAC |
| *OsAUX3* | Forwards： | TTGGTTTAGCTATAGTTGTT |
|  | Reverse： | CTTTTTTTCTGTTCGTTTTT |
| *OsAUX4* | Forwards： | CATTATTGCACTAGTTTTTA |
|  | Reverse： | TTCTCACTCTCACTCTCTCT |
| *OsAUX5* | Forwards： | CGAGCACGGTCAACTTC |
|  | Reverse： | CTAGTGTCTTGGAGGGC |
| *OsCAT1* | Forwards： | TGGTACATTGATGCGAAGA |
|  | Reverse： | TACATGACACGACAGACGG |
| *OsCAT7* | Forwards： | AACTTCAGACTCTGGGTATT |
|  | Reverse： | CTTTCTGTCGCCTTATCT |
| *OsBAT1* | Forwards： | CACCCTCAACTACACCCCC |
|  | Reverse： | CTGCATTTCCATATCCACC |
| *OsBAT7* | Forwards： | CTTGGTGCTGTGCTTGGTT |
|  | Reverse： | GATGAAGGTTAGGCCGATGTA |
